# Cancer cachexia: A scoping review on non-pharmacological interventions

**DOI:** 10.1016/j.apjon.2024.100438

**Published:** 2024-03-12

**Authors:** Elisabetta Bertocchi, Francesco Frigo, Loredana Buonaccorso, Francesco Venturelli, Maria Chiara Bassi, Silvia Tanzi

**Affiliations:** a Palliative Care Unit, Azienda USL-IRCCS di Reggio Emilia, Italy; b Gastroenterology Unit, Città della Salute e della Scienza di Torino, Turin, Italy; University of Turin, Torino, Italy; c Psycho-Oncology Unit, Azienda USL-IRCCS di Reggio Emilia, Italy; d Epidemiology Unit, Azienda USL-IRCCS di Reggio Emilia, Italy; e Medical Library, Azienda USL-IRCCS di Reggio Emilia, Italy

**Keywords:** Cachexia, Neoplasm, Systematic review, Psychosocial intervention, Palliative care, Quality of life

## Abstract

**Objective:**

Cancer cachexia occurs in 30%–80% of patients, increasing morbidity and mortality and impacting the health-related quality of life also for caregivers. Pharmacological interventions have been studied but have shown inconsistent effects on patients' lives in terms of relative outcomes and poor adherence to pharmacological treatment. We provide an overview of the evidence on non-pharmacological interventions for cancer cachexia.

**Methods:**

We conducted a scoping review based on Preferred Reporting Items for Systematic Reviews and Meta-Analyses-extension for scoping review (PRISMA-ScR). On September 21, 2022, plus an update on January 10, 2024, we searched MEDLINE, Embase, CINAHL, Cochrane, PsycINFO, and Scopus for 2012–2024. We excluded pharmacological interventions defined as “any substance, inorganic or organic, natural or synthetic, that can produce functional modifications, through a chemical, physicochemical or physical action.”

**Results:**

The search retrieved 9308 articles, of which 17 were eligible. Non-pharmacological interventions included nutritional counseling, complementary therapies (acupuncture), rehabilitation, and psychoeducational/psychosocial support. The data showed small and heterogeneous samples and different disease localization and stages. Thirty-nine percent were multimodal interventions and aimed at patients, not families. The common primary outcomes were body weight and composition, biomarkers, quality of life, psychological suffering, and muscular strength. Only three studies focus on the patient-caregiver dyad.

**Conclusions:**

Interventions on cancer cachexia should be multimodal and multiprofessional, proposed early, and aimed at quality of life outcomes. The caregiver's involvement is essential. Nurses can play an active role in managing cancer cachexia. More well-designed studies are needed to understand the efficacy and contents of non-pharmacological interventions.

**Systematic review registration:**

The review protocol has been registered in the OSF registry (DOI: 10.17605/OSF.IO/H4A29).

## Introduction

Cancer cachexia occurs in 30%–80% of patients, and its impact on quality of life, treatment-related toxicity, physical function, and mortality is well established.^1^ The European Society for Medical Oncology (ESMO) Clinical Practice Guidelines^2^ define cachexia as disease-related malnutrition, based on the Global Leadership on Malnutrition (GLIM) definition,^3^ and the presence of systemic inflammation. Cancer cachexia is a continuum with three stages of clinical relevance: precachexia, cachexia, and refractory cachexia.^4^ It includes “objective” components (i.e., inadequate food intake, weight loss, inactivity, loss of muscle mass and metabolic derangements, inducing catabolism) and “subjective” components (i.e., anorexia, early satiety, taste alterations, chronic nausea, distress, fatigue and loss of concentration).^2^^,^^5^

Cancer cachexia alters appearance, affecting the patient's self-image, self-esteem, and socialization.^6^ Additionally, it impacts family functioning regarding the role and the meaning of food in the relationship of the patient-caregiver dyad.^6^ In particular, in the last weeks of life, the inability to eat/drink and body image changes can result in emotional distress for the dyad.^6^, ^7^, ^8^ For these aspects, psychosocial interventions such as education, dietary advice, and emotional counseling are proposed,^2^^,^^8^ which reduce the emotional burden by empowering dyads to cope with the dysfunctions and derangements of cachexia, thus improving their quality of life.^7^ Tailored information according to the stage of cachexia also empowers the dyad to understand its nature, course, and biological mechanisms and to acknowledge its adverse effects (i.e., weight loss, reduced appetite, early satiety).^2^

Comprehensive treatment requires a personalized and multidisciplinary approach to evaluate the objective signs and relieve the symptoms.^2^^,^^9^ Core component interventions should thus include nutritional support and exercise-based, anti-inflammatory, and educational interventions.^2^^,^^9^ Pharmacological interventions are widely studied, but evidence-based practice has shown that it is difficult for patients to comply with the intake of supplements and non-steroid anti-inflammatory drugs, which are the most abandoned components, especially among patients in palliative care, where a 20% dropout rate has been seen.^10^^,^^11^ Furthermore, this intervention alone cannot respond to the many aspects affected by cachexia.

Although the literature regarding non-pharmacological components is growing, the limited evidence is acknowledged by the international guidelines.^12^ Studies on psychoeducational approaches to support patients and their families are becoming more common. A scoping review conducted in 2023 explored the extent to which nurse-led education has become part of the multimodal management of cancer cachexia. Nine publications were included in the review. The findings showed that nurses with the knowledge and confidence to provide cancer cachexia education for their patients could potentially play an essential role in the management of cancer cachexia and the mitigation of cachexia-related problems.^13^ Physical exercise can reduce the effects of cancer cachexia by modulating muscle metabolism, reducing insulin resistance, and decreasing the inflammatory cascade. A scoping review conducted by Canaan Cheung et al., in 2023 included 12 randomized and non-randomized studies, concluding that exercise interventions appear to be safe and acceptable to people with cancer cachexia. They could have a positive effect on body stature (weight and body mass index [BMI]), composition (75%), muscle strength (80%), and less often observed for functional performance (64%) and health-related quality of life (38%).^12^

In the specific population of patients with an expected survival of less than a few months, comfort-directed care is the recommended approach, including alleviating thirst, eating-related distress, and other debilitating symptoms.^2^^,^^10^ This includes addressing dysfunctions associated with the emotional and social aspects of eating and involving caregivers.

In conclusion, a non-pharmacological approach can contribute to filling the gap caused by low adherence to pharmacological interventions, and it is particularly important in managing both clinical aspects and supporting the emotional distress of the dyad, in particular with patients who have a short life expectancy. Non-pharmacological interventions can also be delivered by trained health professionals such as nurses and physiotherapists, who thus play a crucial role in the multidimensional management of cancer cachexia.

Considering these premises, we decided to provide an overview of the available research evidence on non-pharmacological interventions for cancer cachexia.

Our primary research question was: Which non-pharmacological interventions have been studied for managing cancer cachexia?

## Methods

This systematic review was conducted following the Preferred Reporting Items for Systematic Reviews and Meta-Analyses-extension for scoping review (PRISMA-ScR) guidelines.^14^ Ethical approval was not required. The protocol was published on https://osf.io/registries (OSF Registration DOI: 10.17605/OSF.IO/H4A29) on December 16, 2021.

### Search strategy

We conducted an electronic search of the literature on September 21, 2022, and one update on January 10, 2024, in the following databases: MEDLINE (through PubMed), Embase, Cochrane Library, CINAHL, PsycINFO, and Scopus. We limited the search to the last 12 years (2012–2024) because the topic has recently been studied more in depth, especially concerning non-pharmacological interventions, and we included only articles in English and articles on humans. The search strategy used was: (Cachexia OR anorexia OR “Cachexia” [Mesh] OR “Anorexia” [Mesh]) AND (cancer OR tumor OR neoplasm OR oncol∗ OR “Neoplasms” [Mesh]). No additional searching was conducted, but we screened the references of the included articles for any additional relevant articles.

### Inclusion criteria

The PICO(S) (Population, Intervention, Comparison, Outcomes, Study design) framework was used to frame the search strategy and to define the inclusion criteria.

Concerning the population, we included original studies with adult human patients (> 18 years) with cancer (regardless of disease location or stage and regardless of ongoing or planned treatment) in which at least 70% of enrolled patients were affected by cancer cachexia. Starting with Fearon's shared definition of cancer cachexia,^4^ we tried to take a more comprehensive look at the eligible population. Therefore, we included studies where the description and characteristics of the sample clarified the inclusion of patients with significant weight loss or high risk of malnutrition. The eligible interventions (i.e., “non-pharmacological interventions”) were defined starting from the definition of “pharmacological intervention” as “any substance, inorganic or organic, natural or synthetic, that can produce functional modifications, through a chemical, physicochemical or physical action.” Based on this definition, we considered “pharmacological intervention” not only medical drugs but also oral nutritional supplements, enriched food, and parenteral and enteral nutrition. Interventions other than those defined as “pharmacological” were defined as “non-pharmacological” and were considered eligible for our review. In the case of randomized controlled trials, we also considered eligible studies where the non-pharmacological component was in the control arm. We also included studies on multimodal interventions, where the pharmacological component was integrated with the non-pharmacological one, but only if the results related to the pharmacological component were residual.

The eligible comparators, when applicable, were pharmacological interventions, placebo, or usual care.

Reported outcomes were related to quality of life, psychological outcomes, muscle strength tests, body composition, and nutritional biomarkers. Since the review aimed to report the non-pharmacological interventions described in the literature rather than assess their efficacy, outcomes were not considered inclusion criteria. Outcomes were described when available in the included studies to provide information on how non-pharmacological interventions were evaluated in the literature.

For the same reason, the study design was not considered as part of the inclusion criteria but was reported to provide information on non-pharmacological intervention assessments published in the literature.

### Exclusion criteria

We did not include conference abstracts, case reports, qualitative studies, systematic reviews, expert opinions, descriptive articles, guidelines, or book chapters. Lastly, we excluded ongoing trial and protocol articles.

### Study selection process

**Fig. 1:**
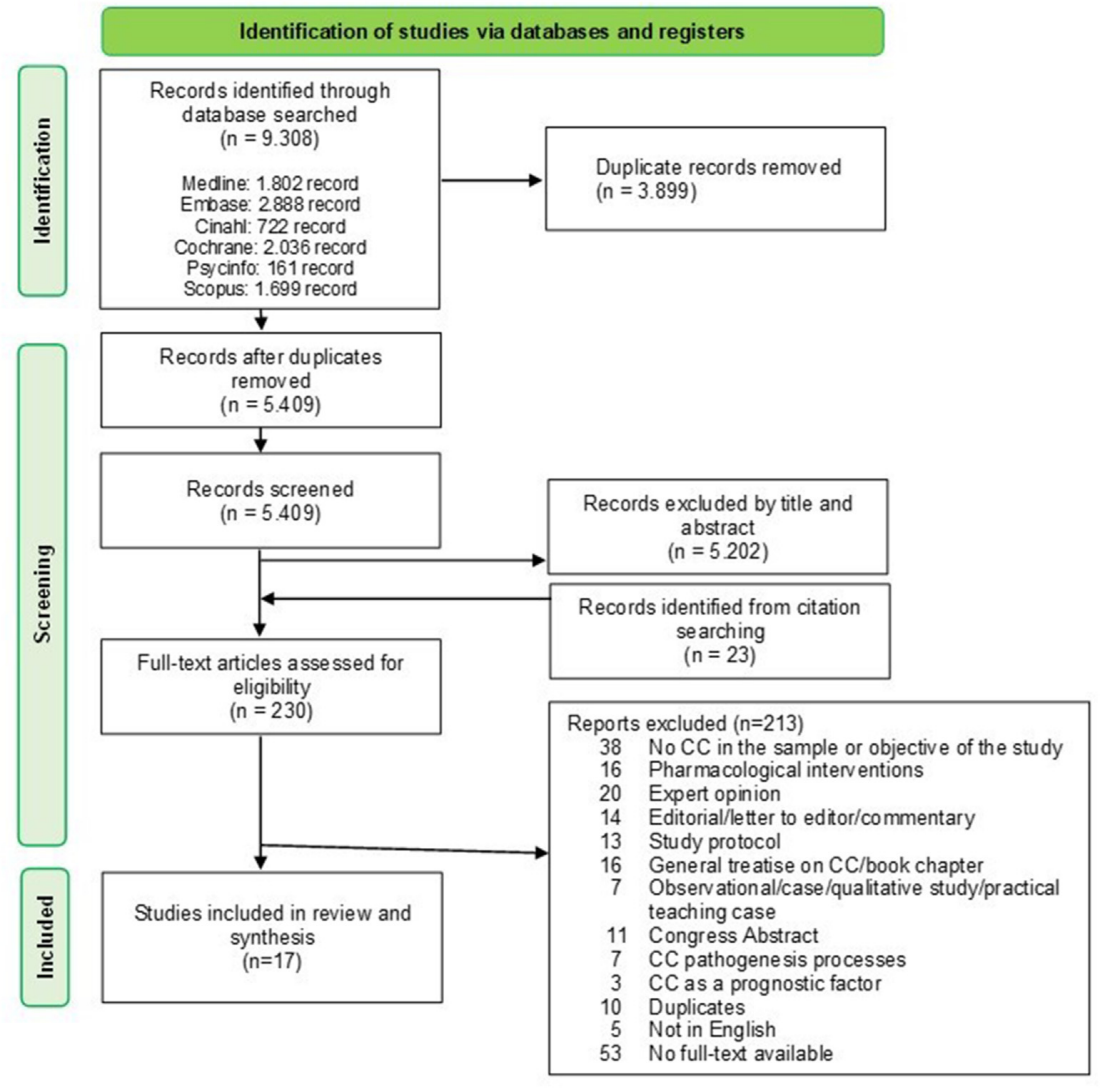
Scoping review process.

The results of the study selection process are reported in Fig. 1. Three authors (EB, LB, and FF) independently assessed the titles and abstracts of the articles included in the first search. Then, they discussed all the discrepancies and doubts with a fourth researcher (ST). In all cases, they reached a consensus. The same procedure was carried out for the analysis of the full text. If all three authors (EB, LB, and FF) agreed that the studies met the eligibility criteria, these were included in the results, and any disagreements were discussed and resolved by consensus with a fourth author (ST).

### Data extraction

Two reviewers (EB and FF) performed data extraction from included full-text articles using a data extraction form developed by the working group. The data extraction form included the first author's name, year of publication, country, study design, inclusion criteria, exclusion criteria, study population, tumor characteristics (site + stage), cancer treatments, reference definition of cachexia, description of the intervention, description of comparison (when applicable), follow-up time, adherence, drop-out reasons, and reported outcomes.

### Risk of bias assessment

Two authors (LB and ST) independently performed the quality/risk of bias assessment of the included studies. The third expert methodology member of the research group (FV) appraised the data as a supervisor, using different tools according to the different study designs. The Cochrane Risk of Bias tool 2 (RoB2) tool was used to assess the included randomized trials,^15^ while the Newcastle-Ottawa Scale^16^ was used for non-randomized studies. The results of the risk of bias assessment are reported in detail, separately by study design (i.e., randomized and non-randomized studies), in the Supplementary material (Supplementary File 1 with figures, Supplementary Files 2 and 3 with tables). The risk of bias graph and the risk of bias summary figures were built with Review Manager, using the Web version (https://revman.cochrane.org/info).^17^

### Synthesis of the results

As the aim of the scoping review was not to evaluate the effectiveness of non-pharmacological interventions, a meta-analysis of the results was not included in the research protocol. We therefore included the possibility of a quantitative and qualitative narrative synthesis of results. We have included an additional table and a figure summarizing the results to speed up the reading and identify the articles of interest.

## Results

The search retrieved 9308 records, which were reduced to 5409 records after removing duplicates. After the initial screening by title and abstract, another 5202 articles were excluded. Of the 230 full-text articles retained for further screening, 213 were discarded. Most of the studies were excluded because the sample did not include patients with cancer cachexia, or they were the smallest part. Other articles were excluded because of editorials, commentaries, or general treatises on cancer cachexia. Seventeen articles were included in the full review (Fig. 1).

These articles correspond to 15 original studies because, in 2015 and 2019, Grundmann and Yoon wrote two articles on the same studies, respectively, on the prospective feasibility pilot study^18^^,^^19^ and the randomized, single-blind pilot study.^20^^,^^21^ The articles cover five continents: five studies conducted in Europe,^22^, ^23^, ^24^, ^25^, ^26^ two in North America,^18^^,^^19^ four in Asia,^27^, ^28^, ^29^, ^30^ one in Africa,^31^ one in Australia,^32^ and one in two different sites in Australia and Hong Kong.^33^ Overall, the randomized controlled trial was the most common study design, accounting for 58% of the studies.^20^, ^21^, ^22^, ^23^, ^24^^,^^27^^,^^29^, ^30^, ^31^^,^^33^ Other authors chose pre-post intervention design (*n* = 2),^18^^,^^19^ retrospective observational study (*n* = 2),^32^^,^^34^ prospective cohort study (*n* = 1),^25^ and prospective pilot study.^26^

The results showed heterogeneity in populations and interventions, most of which were multimodal. The non-pharmacological components, alone or combined with others, were nutritional counseling, complementary therapies (e.g., acupuncture), exercise, and psychoeducational/psychosocial interventions. The most common primary outcomes were body weight and body composition, biomarkers, quality of life, psychological suffering, and muscular strength (Table 1).

**Table 1:** Characteristics of the included studies based on PICO criteria (patients, intervention, study design, comparison, patients, outcomes).

No.	Author, year Country	Study design	Population	Intervention group	Type of comparison	Follow-up time	Indicators/Outcomes	In favor of
**1**	Hopkinson, 2010 United Kingdom	Cluster-randomized design, with two community palliative care teams randomized to different arms	65 patients with advanced cancer, and concerns about patient's weight and eating	*N* = 35 Support offered by MAWE-trained clinical nurse specialists, during home consultation, between patient, caregiver, and nurse. Leaflets “Living with Changes in Eating”	*N* = 30 Usual care	Not declared	Interviews pre-and post-intervention for deliverability, acceptability, and patient-perceived effect of MAWE; VAS for self-reported eating-related distress (ERD) and weight-related distress (WRD).	Macmillan Approach to Weight and Eating (MAWE) intervention is deliverable by trained nurses and acceptable to patients; it can mitigate WRD and ERD in people with advanced cancer.
**2**	Faber, 2015 Netherlands	Exploratory double-blind placebo-controlled RCT	64 newly diagnosed esophageal cancer patients	*N* = 31 4-week nutritional intervention: Dietary counseling + active medical food (nutritionally complete oral supplement)	*N* = 33 4-week nutritional intervention: Dietary counseling and advice + For group 0–5% WL, a non-caloric placebo product. For group ≥ 5% WL, an iso-caloric standard nutritional product	After 2 weeks and after 4 weeks	Primary outcome: Markers for immune function. Secondary outcomes: Body weight, Eastern Cooperative Oncology Group performance status (ECOG PS), white blood cell and lymphocyte subset count, inflammatory cytokines, serum prostaglandin (PGE_2_), phospholipid fatty acids, pre-albumin, albumin, QoL, dysphagia.	No differences, between intervention group (IG) and control group (CG) were observed on the change from baseline regarding primary outcome. Nutritional intervention (IG) with the specific medical food significantly increased body weight and improved performance status. This effect was accompanied by significantly reduced serum PGE2 levels.
**3**	Focan, 2015 Belgium	Prospective RCT feasibility study	53 cancer patients with cachexia treated for cancer	*N* = 27 Standard management of cachexia + psychological and dietetics workshops offering a cognitive-behavioral approach based on full-body mindfulness philosophy	*N* = 26 Standard management of cachexia: Standard dietetics support; eventual nutritional complements according to estimated patient needs	T1: After 1 month (or 2 × 2 workshops) T2: After 2 months (or 4 × 2 workshops)	Detailed quantitative and qualitative food anamnesis. Quality of life: EORTC QLQ-C30 Mindfulness approach: FFMQ Satisfaction questionnaire Body weight, BMI, total daily calories intake, WHO score	The experimental group showed a significant benefit in body weight, BMI, WHO status score, emotional function, fatigue, digestive disorders, and faculty of observation. Satisfaction questionnaires: Positive appreciation of workshops with a satisfaction rate of 75%.
**4**	Grundmann, 2015 USA	Prospective feasibility pilot study Pre- and post-intervention single group design	7 patients with gastric or colorectal cancer, undergoing chemotherapy	Acupuncture	Not applicable	After at least four BIA measurements	BIA measurements (total body water, extracellular fluid, intracellular fluid, fat mass, fat-free mass, BMI, phase angle, % weight change)	Acupuncture is acceptable and it may be able to reduce or halt the progression of weight loss and preserve a normal metabolism. BIA is promising to evaluate the health status of patients.
**5**	Yoon, 2015 USA	Prospective feasibility pilot study Pre- and post-intervention single group design	7 patients with gastric or colorectal cancer, undergoing chemotherapy (same population as Grundmann et al., 2015)	Acupuncture	Not applicable	After at least four BIA measurements	VAS for appetite, Simplified Nutritional Appetite Questionnaire (SNAQ), Karnofsky PS, BIA Participants were also asked about their expectations of the study.	Acupuncture seemed to improve appetite and slow weight loss in patients with GI cancers. All participants were very optimistic about the acupuncture intervention and expected outcomes, which were noted prior to the intervention.
**6**	Kapoor, 2017 India	Prospective RCT	63 female patients with advanced cancer attending palliative clinics, with symptoms of cachexia	*N* = 30 Nutritional and physical activity counseling + daily nutritional supplementation of 100 g of IAtta (IG) for 6 months.	*N* = 33 Nutritional + physical activity counseling	At 3 months and post-intervention (6 months)	Anthropometric parameters (body weight, mid-upper-arm circumference (MUAC), body fat); nutritional status parameters (dietary intake: Energy, carbohydrate, protein, fat), PG-SGA; physical activity Level (Indian Migrant Study Physical Activity Questionnaire IMS-PAQ); EORTC QLQ-C30 (global health status, social functioning, fatigue, pain, appetite loss)	In the IG, patients had better control of cachexia symptoms, maintaining or improving the anthropometric, nutritional, and QoL parameters. On the contrary, in the CG, patients worsened with respect to the measured parameters. Embedding nutrition supplementation within the palliative care therapy may improve quality of life and stabilize body weight in cancer cachexia patients.
**7**	Parmar, 2017 Canada	Retrospective study	374 patients with advanced cancer, attending the McGill Cancer nutrition rehabilitation program clinic at the Jewish general hospital (CNR-JGH) and suffering weight loss, anorexia, or generalized functional decline.	All patients attended the clinic. The clinic team was composed of a physician, nurse, physiotherapist, and dietitian. Patients were evaluated by each professional at each visit and an inter-disciplinary intervention plan was formulated.	Not applicable	After the second visit (6 weeks) and after the third visit (12 weeks)	Retrospective chart review, for - FAACT score (total and physical, emotional, functional, anorexia-cachexia subscales) - Trial outcome index (TOI), which comprises physical, functional, and cachexia subscales of FAACT - 6 min walk distance test (6 MWT) Further analysis was performed to establish which clinical features were associated with differences in QoL total score and TOI at visit 1.	The multimodal approach offered by the CNR-JGH results in clinically important improvements in QoL. All patients who are able to receive this type of intervention have similar potential to improve their QoL, but the greatest benefits are seen in those who gain weight and improve their 6 MWT.
**8**	Grundmann, 2019 USA	Randomized single-blind pilot study	38 gastrointestinal cancer patients under chemotherapy (no radiotherapy or surgery)	*N* = 20 Targeted acupuncture (TAG)	*N* = 18 Non-targeted acupuncture (NTA)	Baseline and then weekly until interval completion (8 weeks)	- Body composition Bioelectrical impedance analysis (fat-free Mass FFM, Intracellular Water ICW, Extracellular water ECW) - Biomarkers Appetite hormones (leptin, ghrelin), systemic inflammation (CRP, TNF-α), nutritional status (prealbumin), and LDH	While both groups maintained their weight, the TAG demonstrated a trend in weight gain in weeks 7 and 8
**9**	Yoon, 2019 USA	Randomized single-blind pilot study The same study as Grundmann, 2019	38 gastrointestinal cancer patients under chemotherapy (no radiotherapy or surgery)	*N* = 20 Targeted acupuncture (TAG)	*N* = 18 Non-targeted acupuncture (NTA)	Baseline and then weekly until interval completion (8 weeks)	Secondary outcomes: Gender differences across and within TA and NTA groups, with respect to outcome measures described in Grundmann, 2019.	Results, even if often non-significant, suggest a gender-specific response, probably based on hormone-specific regulation of food intake.
**10**	Yuliatun, 2019 Indonesia	Exploratory and experimental study with pretest-posttest design	7 Breast cancer patients (stage 2-3-4); no treatments during the intervention	Manual acupuncture: Eight sessions, every two days	Not applicable	At baseline and at the end of the intervention (after 8 sessions)	Body composition: Body weight, BMI, fat mass (FM), and fat-free mass (FFM), through BIA	Acupuncture is well tolerated and feasible in breast cancer patients with cachexia. Body weight, BMI, and fat-free mass remained almost stable after the 8 acupuncture sessions, suggesting prevention of the progression of muscle wasting.
**11**	Kamel, 2020 Egypt	Single-blind RCT	40 patients with pancreatic cancer and cancer-induced cachexia	*N* = 20 resistance training group An exercise regimen, twice a week for 12 weeks, supervised by specialized physical therapists.	*N* = 20 Control group Nutritional and psychosocial support	Baseline, and 12 weeks after intervention	Mobility, muscle strength, and lean body mass	Three months of a resistance training program in patients with pancreatic cancer-induced cachexia led to improvement in mobility and isokinetic and isometric muscle strength, with significant outcomes for some muscle groups.
**12**	Latenstein, 2020 Netherlands	Prospective multicenter cohort study	202 patients with pancreatic and periampullary cancer	Dietetic consultation Tube feeding Oral nutritional supplement	Not applicable	Baseline and at 3, 6, 9, 12, 18, and 24 months after baseline, and yearly thereafter, until death or dropout.	Overall survival. PROMs questionnaire data included self-reported nutritional parameters and body weight (www.pacap.nl). Nutritional status: Height, current weight, weight loss, dietetic consultation (including both intramural and extramural health care), self-reported reduced food intake, appetite, use of oral nutritional supplements or parenteral nutrition, and tube feeding. BMI (kg/m^2^). Dietary intake: By the Dutch Healthy Diet Food Frequency Questionnaire (DHD-FFQ).	Higher prevalence of dietetic consultations in cachectic patients undergoing best supportive care (71%) compared with patients undergoing palliative chemotherapy (52%) or surgery (53%). Increased awareness of cachexia and severe weight loss, screening on (the risk of) malnutrition based on the GLIM criteria, and dietetic consultation to improve protein intake could be helpful in improving treatment outcomes.
**13**	Bland, 2021 Australia	Retrospective observational review	162 cancer patients, attended three times the Barwon Health Cachexia and Nutritional Support Service in Geelong, Victoria, Australia, between 2017 and 2020.	A multidisciplinary, multimodal approach to cancer cachexia care. The care team includes a palliative medicine physician, nurse practitioner, dietitian, and physiotherapists.	Not applicable	At the first visit, second visit (1-month follow-up), third visit (3-month follow-up)	EORTC QLQ-C15-PAL FAACT questionnaire.	Compared to the maintenance of weight and muscle strength, the data show a statistically significant improvement in almost all quality of life and FAACT outcomes over time.
**14**	Molassiotis, 2021 Australia and Hong Kong	Non-blinded pilot RCT	74 advanced cancer patients and family caregivers, attending the ambulatory at the Royal Brisbane and Women’s Hospital (Australian site), and Haven of Hope Hospital and Shatin Hospital (Hong Kong site).	*N* = 34 A family-centered nutritional intervention is conducted by a dietitian. The intervention was composed of three structured sessions (2–3 h) over a 4-week period, including telehealth or telephone follow-ups.	*N* = 40 Usual care	At baseline and at the third scheduled session.	Feasibility (recruitment, consent rate, retention rate, acceptability of assessment tools). For patients • Quality of life: FAACT scale • Nutritional status: PG-SGA-SF (Short Form) 3-day food diary, weight • Eating-related distress: Two single-item checklists, on a 1–10 scale For caregivers • Anxiety and depression: 14-Item HADS • Self-efficacy: 21-Item caregiver self-efficacy scale (CaSES) • Caregiver distress: 18-Item Caregiver distress checklist • Eating-related distress: 19-Item eating-related distress checklist	Good intervention fidelity by patients and caregivers. The assessment tools used were generally acceptable. In both sites, results showed small improvements in IG, in particular in terms of eating-related distress and FAACT QoL for patients.
**15**	Sim, 2022 South Korea	RCT	58 gastrointestinal cancer patients	*N* = 31 The IG received an oral nutritional supplement (ONS) enriched with omega-3 fatty acids	*N* = 27 The CG received nutritional counseling and education	At baseline, after 4 and 8 weeks.	Nutritional status Body composition through BIA, PG-SGA, body temperature, triceps skinfold thickness, and mid-arm muscle circumference, 3-day 24-hour recall, and concurrent dietary records. Quality of life EORTC QLQ-C30 scale. Nutritional biomarkers Blood samples were collected at each visit, analyzing hemoglobin, albumin, prealbumin, cholesterol, serum concentrations of tumor necrosis factor-alpha (TNF-α), interleukin-6 (IL-6), and interleukin-8 (IL-8).	Both groups had an improvement in nutritional status, statistically significant only in the IG.
**16**	Bagheri, 2023 Iran	RCT	46 patients with diagnosis of colorectal cancer in stage 2–4 based on TNM UICC 2010 system	*N* = 23 For IG, a Mediterranean diet regime with extra virgin olive oil was prescribed	*N* = 23 CG received nutritional instructions with dietary recommendations	At the start and end of the study (eight weeks)	Primary outcomes: Muscle strength, lean body mass, nutritional status (PG-SGA) and inflammatory markers (hs-CRP, IL-6, TNF-α). Secondary outcomes: Quality of life (EORTC QLQ-C30), serum albumin and total protein, weight, body fat mass and percent body fat (through BIA).	The findings of RCT showed that a Mediterranean diet rich in extra virgin olive oil led to an improvement in nutritional status, quality of life, inflammatory markers, and body composition.
**17**	Buonaccorso, 2023 Italy	Prospective mixed-method pilot study	24 cancer patients with the presence of cachexia and refractory cachexia, and malnutrition and their caregivers	*N* = 24 dyads received bimodal intervention, including a psycho-educational component and exercises.	NA	T0 (baseline) T1 (two weeks after T0) T2 (four weeks after T0) T3 (eight weeks after T0)	Primary outcome: Completion rate and adherence to both interventions Secondary outcomes: Quality of life FAACT, caregiver burden (Zarit Burden Scale), upper and lower limb physical performance (hand-grip strength test); acceptability of the intervention (semi-structured interviews).	Study findings strongly support the acceptability of the bimodal intervention but only partially support its feasibility.

MAWE, Macmillan Approach to Weight and Eating; VAS, visual analog scale; RCT, randomized controlled trial; WL, weight loss; PS, performance status; QoL, Quality of Life; IG, intervention group; CG, control group; EORTC QLQ-C30, EORTC Quality of Life Questionnaire Core 30; FFMQ, Five Facet Mindfulness Questionnaire; BMI, body mass index; BIA, bioelectrical impedance analysis; PG-SGA, Patient-Generated Subjective Global Assessment; GI, gastrointestinal; FAACT scale, Functional Assessment of Anorexia/Cachexia Therapy scale; EORTC QLQ-C15-PAL, EORTC Quality of Life Questionnaire Core 15 Palliative Care; HADS, Hospital Anxiety and Depression Scale; NA: not available.

**Fig. 2:**
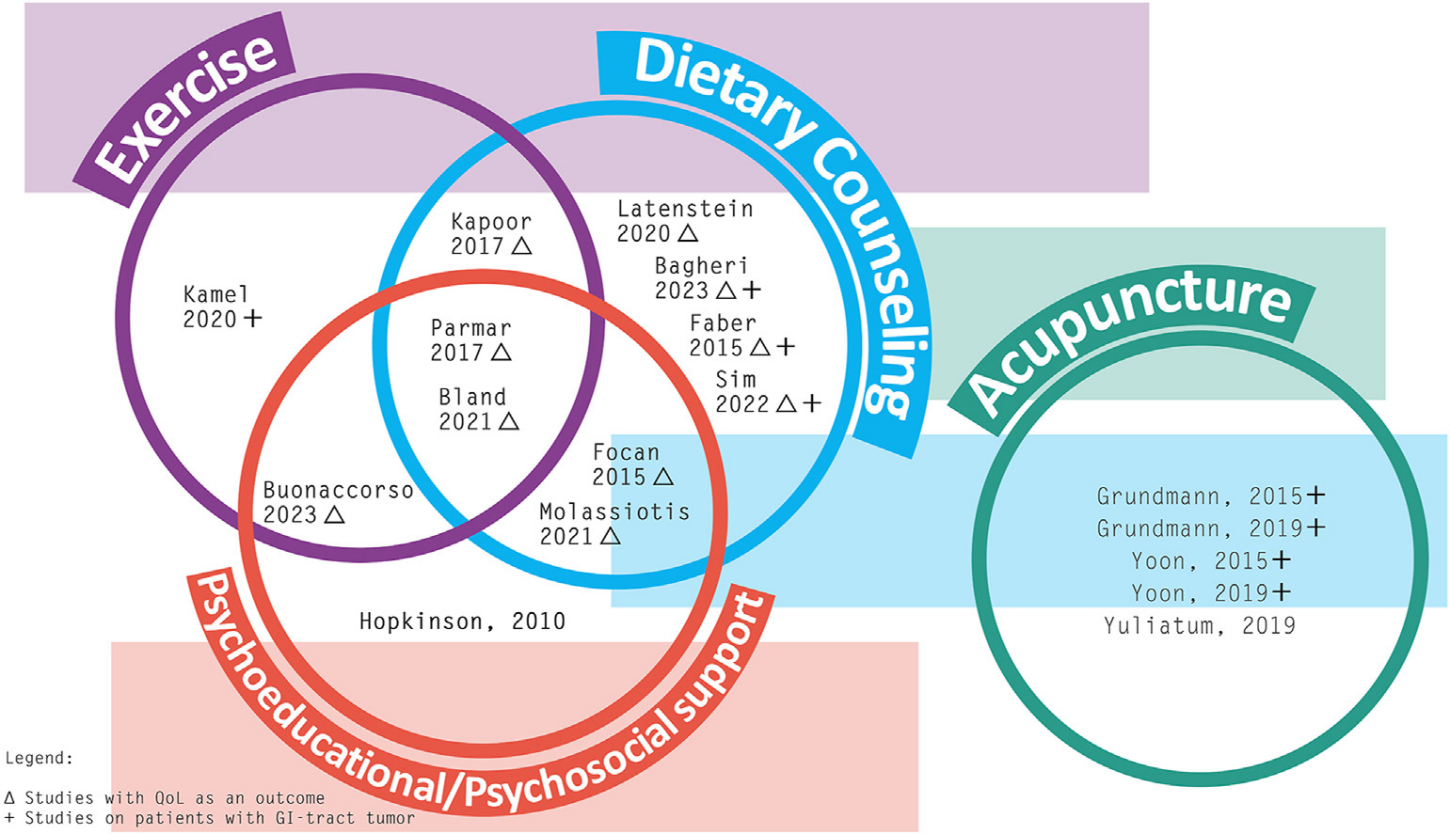
The highlighting characteristics of the interventions.

In Fig. 2, we summarized the articles included in the scoping review, highlighting three characteristics: the multimodal structure of intervention, the presence of quality of life among the outcomes, and the population exclusively composed of patients with gastrointestinal cancer.

Table 2 shows a schematic representation of the selected studies based on patient/population, intervention, comparison -where present- and outcomes (PICO) elements for review. We described the narrative synthesis according to PICO.

**Table 2:** Summary of the main characteristics of the selected articles.

Author, year of publication	Study design		Population			Intervention					Outcome
	RCT	Other design	Only GI∗ cancer	Under cancer treatment	Patient-caregiver dyad	Psychosocial support	Dietary counseling	Exercise	Acu-puncture	Adherence ≥ 70%^§^	QoL∗ among outcomes
Hopkinson, 2010	√			√	√	√				√	
Faber, 2015	√		√				√			√	√
Focan, 2015	√			†		√	√				√
Grundmann, 2015		√	√	√					√	√	
Yoon, 2015		√	√	√					√	√	
Kapoor, 2017	√			†			√	√			√
Parmar, 2017		√		√		√	√	√		n.a.	√
Grundmann, 2019	√		√	√					√	√	
Yoon, 2019	√		√	√					√	√	
Yuliatun, 2019		√							√	√	
Kamel, 2020	√		√					√		√	
Latenstein, 2020		√	√	√			√			n.a.	
Bland, 2021		√		†		√	√	√		n.a.	√
Molassiotis, 2021	√			†	√	√	√				√
Sim, 2022	√		√	√			√				√
Bagheri, 2023	√		√	√			√			√	√
Buonaccorso, 2023		√		√	√	√		√			√

GI, Gastrointestinal; QoL, Quality of life. †, Not declared. §, In the non-pharmacological group. n.a., Not applicable.

### The study population

The results of the review showed small and heterogeneous samples (Table 3).

**Table 3:** Population of the studies selected.

Author, year of publication	Inclusion criteria	Exclusion criteria	Sample characteristics	Tumor characteristics (site + stage)	Under cancer treatments	Reference definition of cachexia
Hopkinson, 2010	(1) Advanced cancer (no longer receiving potentially curative treatment) (2) Patient or family/caregiver concerned about the patient's weight/eating (3) Able and willing to participate (4) Age 18 years or older (5) Nurse-led management (6) No clinical reason for exclusion (nurses made this judgment) (7) 24 h to consider participation before the nurse made a first assessment home visit.	Not declared	Mean age 69 (33–87 years) Intervention group M/F: 19 (76%)/6 (24%) Malnourished (> 5% loss of body mass in 6 months): 18 (72%) Control group M/F: 19 (76%)/6 (24%) Malnourished (> 5% loss of body mass in 6 months): 17 (68%)	Lung *n* = 17 (34%) Gastrointestinal *n* = 15 (30%) Head and neck *n* = 4 (8%) Prostate *n* = 4 (8%) Gynecological *n* = 2 (4%) Breast *n* = 1 (2%) Other *n* = 7 (14%)	*n* = 9 (18%) of total patients were under palliative chemotherapy	Not declared
Faber, 2015	- Newly diagnosed cancer patients - ≥ 18 years - Planned for cancer treatment	- Life expectancy < 3 months - Planned start of anticancer treatment within 3 weeks - ECOG PS ≥ 2 - Esophagus-related surgery after diagnosis before inclusion - Chemo-radiotherapy in the past 5 years - Altered immune function - Dysphagia score of 4 - Dependency on the tube or parenteral nutrition during the previous 4 weeks - Use of fish oil-containing supplements during the previous 4 weeks - Intolerance or allergy to the study products - Pregnancy or lactation - Dementia or altered mental status	Intervention group Subjects in 0–5% WL: *n* = 13/31 Subjects in ≥ 5% WL: *n* = 18/31 M/F: 24/7 Control group Subjects in 0–5% WL: *n* = 16/33 Subjects in ≥ 5% WL: *n* = 17/33 M/F: 26/7 Total patients Patients ≥ 5% of weight loss in the past 3 months: *n* = 35/64 (54.7%) Patients ≤ 5% of weight loss in the past 3 months: *n* = 29/64 (45.3%) M/F: 50/14 BMI: 25.4 ± 4.1	Adenocarcinoma or squamous carcinoma located in the esophagus or gastroesophageal junction (Siewert-Stein classification type I-III)	No, patients are waiting to start the first line of anticancer treatment (no surgery)	Severe and prolonged malnutrition can lead to cancer cachexia, characterized by progressive, involuntary weight loss, anorexia, asthenia, fatigue, depletion of lipid stores, severe loss of skeletal muscle proteins. (Van Cutsem, 2005; Muscaritoli, 2006)
Focan, 2015	- Unwanted body weight loss ≥ 2% during last month OR ≥ 5% during last 6 months - BMI (weight/size^2^) < 20 - A lowered rate of albumin and pre-albumin - Inflammatory syndrome as measured by CRP titration	Not declared	Intervention group M/F: 5/22 Mean BMI: 20.9 (14–31) WHO score (2 + 3): 86.7% Anorexia grades (2 + 3): 72.2% Control group M/F: 6/20 Mean BMI: 22.8 (17.7–34.2) WHO score (2 + 3): 80.0% Anorexia grades (2 + 3): 40% 8/10 patients in both groups were receiving nutritional support; one patient in each group also benefited from enteral feeding.	Breast, GI tract, head & neck, hematological, non-small cell lung cancer (NSCLC), genitourinary, other Metastases Group A 85.2% Group B 65.4%	Not declared (but probably under cancer treatment)	Tuca, 2013; Argiles, 2011
Grundmann, 2015 and Yoon, 2015	- At least 21 years of age - Medical diagnosis of gastric or colorectal cancer - Weight loss of 5% or more since diagnosis - No surgery within the past month - Not scheduled for radiation therapy for the duration of the trial or the next 3 months - Able to speak or understand English - Able and willing to follow the research protocol	- Does not have chemotherapy scheduled after surgery - Will have radiation therapy alone or in combination with chemotherapy - Has a pre-existing or comorbid disorder that may interfere with the measurements (e.g., HIV, infections, hepatitis, Alzheimer's disorder, movement disorders) - Has burn sites or open and infected wounds - Life expectancy ≤ 3 months	M/F: 3/4 Age range: 34–82 years	Gastric or colorectal cancer	Yes, but only chemotherapy (not surgery or radiotherapy)	Cancer cachexia is a debilitating syndrome of progressive weight loss, anorexia, and decreased lean body mass that commonly affects patients with both early- and late-stage disease. (Kern, 1988) More than half of patients undergoing treatment experience malnutrition, anorexia, and weight loss (Smith et al., 2008), which are independent factors that contribute to a lower survival rate, decreased quality of life, and functional impairment. (Andreyev et al., 1998; Fearon et al., 2011; Tisdale, 2009)
Kapoor, 2017	- Female gender - ≥ 18 years - Weight loss of more than 5% from pretreatment weight - BMI less than 20 kg/m^2^ along with a hemoglobin level less than 12 g/dL - Energy intake of less than 1,500 kcal/d	- Gastrointestinal disorders - On anabolic steroids - Taking synthetic ONSs - Life expectancy ≤ 3 months	Intervention group - Mean weight (kg) 39.7 ± 5.7 - Mean MUAC (cm) 20.8 ± 2.1 - Mean body fat (%) 20.5 ± 5.2 Control group - Mean weight (kg) 41.1 ± 7.3 - Mean MUAC (cm) 22.2 ± 2.4 - Mean body fat (%) 25.4 ± 6.5	Anorectal, bone, brain, breast, buccal cavity, chest wall, eyelid, female genitourinary tract, lung, olfactory, spine, suprarenal mass, thyroid	Not declared (all patients attended palliative Care Clinic for symptom management)	Cachexia progresses through different stages, initiated by less than 5% pretreatment body weight loss along with anorexia and various metabolic changes (i.e., pre-cachexia stage), to more than 5% weight loss with sarcopenia and systemic inflammation (i.e., cachexia stage), and finally becoming unresponsive to anticancer treatment, with less than 3 months of expected patient survival (i.e., refractory cachexia). (Fearon, 2011)
Parmar, 2017	All the patients (*n* = 374) with advanced cancer, attending the McGill Cancer Nutrition Rehabilitation program clinic at the Jewish General Hospital (CNR-JGH) and suffering weight loss, anorexia, or generalized functional decline.	Not applicable	M/F: 208/166 (55.6%/44.4%) Patients with cachexia: 68.7% ECOG PS 1 = 34.5% 2 = 46.5% ≥ 3 = 16.5% Body weight change (mean %): Over prior 6 months −10.2 kg (9.8%); over prior 6 weeks −2.6 kg (5.9%)	Lung, GI tract, hematological, breast, other.	Cancer treatment line (*n* = 184, 49%) 0 = 37.5% 1 = 35.3% 2 = 13.6% ≥ 3 = 13.6%	Cachexia is a debilitating wasting syndrome affecting up to 80% of cancer patients with advanced diseases characterized by involuntary, progressive weight and muscle loss, reduced physical function and associated symptoms such as anorexia and fatigue. (Fearon, 2011; Tisdale, 2009)
Grundmann, 2019 and Yoon, 2019	- Adult patients (≥ 21 years) - Diagnosis of primary GI cancer - Weight loss of at least 5% over the last 6 months - Ability to communicate in English - Ability to follow the research protocol	- Planned to have surgical procedures at the time of recruitment or during the month before the study - Would receive radiation therapy during the study - Underwent surgery during the study or would not have chemotherapy after surgery - Any comorbidity that may affect the interpretation of study findings (e.g., HIV, AIDS, Alzheimer's disease, movement disorder …) - Had open burn sites or infected wounds - Was diagnosed with esophageal cancer with a mechanical swallowing difficulty - Had an uncorrected, mechanical digestive obstruction or inability to tolerate enteral nutrition - Had a diagnosis of pancreatic adenocarcinoma - Life expectancy ≤ 6 months	M/F: 17/21 Age (mean, range): 57.4 (27–76)	Colorectal 27/38 Stage 2–5 Stage 3–4 Stage 4–7 Undetermined - 11 Gastric 7/28 Stage 1 - 1 Stage 3–1 Stage 4 - 4 Undetermined - 1 Biliary 4/28 Stage 4–1 Undetermined - 3	No radiotherapy or surgery, only chemotherapy	Cancer cachexia is characterized by significant weight loss, sarcopenia, and an underlying inflammatory process that often leads to higher morbidity and mortality. (Porporato, 2016) Cancer cachexia is a multifactorial syndrome disorder characterized by progressive unintentional weight loss, decreased lean muscle mass, and derailed physiological functioning affecting appetite, immune system response, quality of life, and gastrointestinal motility. (Porporato, 2016)
Yuliatun, 2019	- Weight loss of at least 5% over the last 6 months - Ability to follow the research protocol - No surgery plans for the next 20 days - Never had chemotherapy AND there are no chemo-radiotherapy plans for the next 20 days	- Diabetes mellitus - Afraid of acupuncture needles and acupuncture procedures	4/7 patients had non-critical weight loss (≤ 10%) in the previous 6 months 3/7 patients had critical weight loss (> 10%) in the previous 6 months BMI (before diagnosis): 25.3 ± 3.6 BMI (at the start of the study): 20.4 ± 2.9	Breast cancer Stage 2: 2/7 patients Stage 3: 2/7 patients Stage 4: 3/7 patients	Neither radio- nor chemotherapy	Cachexia is a wasting syndrome that is a common condition in advanced breast cancer patients and metastasis. (Tuca, 2013)
Kamel, 2020	- Adults ≥ 20 years of age - Weight loss > 5% over the past six months AND weight loss > 2% in patients with a body mass index of less than 20 kg/m^2^	- Any musculoskeletal or neurological disorders - Severely impaired hematological capacity - Uncontrolled hypertension - Heart failure and unknown arrhythmia - Severe renal impairment - Diminished ability to stand or walk OR any other comorbidities that might hinder exercise	Intervention group M/F: 12 (60%)/8 (40%) BMI (kg/m^2^): 21.15 ± 1.45 Control group M/F: 14 (70%)/6 (30%) BMI (kg/m^2^): 21.06 ± 0.81	Resectable or non-resectable pancreatic cancer (stage I to IV)	No, only patients in follow-up (authors described the number of days after surgery or first chemotherapy)	Cachexia is a syndrome with multiple factors characterized by continued depletion of the skeletal muscle mass, with or without a reduction in fat mass, and cannot be reversed with traditional nutritional therapy. (Fearon, 2011)
Latenstein, 2020	All patients that participate in the Dutch Pancreatic Cancer Project (PACAP) (www.pacap.nl)	Patients were excluded if the questionnaire was not completed at baseline	M/F: 108 (53%)/94 (47%) PS 0–1: 132 (65%) PS ≥ 2: 21 (10%) CACHEXIA at diagnosis Total patients 144 (71%) Surgery patients 59 (63%) Palliative chemotherapy patients 54 (77%) BSC patients 31 (82%) At baseline, 40% of total patients presented ≥ 10% weight loss during the past 6 months	Pancreatic and periampullary cancer	47% surgery (61% neoadjuvant and/or adjuvant chemotherapy) 35% palliative chemotherapy 19% best supportive care	Cachexia is defined as weight loss greater than 5% or weight loss greater than 2% in individuals with a low BMI (BMI < 20 kg/m^2^) or low skeletal muscle mass (sarcopenia) during the past 6 months. (Fearon, 2011)
Bland, 2021	- Age 18 years or over - confirmed diagnosis of cancer - Attended the clinical service at least three times between March 2017 and May 2020 - Able to communicate in English	There were no exclusion criteria.	M/F: 94 (58%)/68 (42%) AKPS ≥ 70%: 70% Mean BMI: 23.1 ± 4.7 Mean weight loss over the previous six months was 10.4% ± 9.4%. Cachexia stage: No cachexia 28 (17%) Pre-cachexia 7 (4%) Cachexia 83 (51%) Refractory cachexia 29 (18%) Unknown 15 (9%) PG-SGA: Stage A (well-nourished) 9 (6%) Stage B (moderately/suspected of being malnourished) 87 (54%) Stage C (severely malnourished) 38 (24%) Unknown 28 (17%)	Disease stage: Metastatic 120 (74%) Locally advanced 42 (26%) Tumor type: Upper gastrointestinal 49 (30%) Lung 38 (23%) Colorectal 24 (15%) Prostate 17 (11%) Head and neck 8 (5%) Other 26 (16%)	Not described.	Cancer cachexia is a complex, multifactorial syndrome that affects an estimated 50% of all people diagnosed with cancer, including up to 80% of those with advanced disease. (Von Haeling, 2016 and 2014; Vagnildhaug, 2018) Cachexia is characterized by the ongoing loss of skeletal muscle mass (with or without the loss of fat mass) that leads to progressive functionalI mpairment. (Fearon, 2011)
Molassiotis, 2021	For patients: - Aged ≥ 18 years old - Stage III or IV cancer - With a life expectancy of ≥ 6 months in the opinion of the treating medical oncologist - At risk of malnutrition from any cause (≥ 2 assessed by the MUST) - Capable of oral food intake - ECOG score 0–2 - Living at home with a caregiver (Hong Kong sites) and with or without a caregiver (Australian site) - Able to communicate in English (Australian site) or Chinese (Hong Kong sites) and complete the study questionnaires with or without assistance For caregivers: - Aged ≥ 18 years old - A family member (e.g., husband/wife, children, relatives), or someone who is designated to take care of the patient and who visits for at least 1 h per day on most days - Able and willing to provide regular assistance with meals and/or nutritional support at home (ideally being present for 2 or more meals each day) - Able to communicate in English (Australian site) or Chinese (Hong Kong sites) and fill in the study questionnaires.	- Completely nil by mouth or participating in other types of nutrition intervention research or receiving enteral/parenteral nutrition - Unable to give informed consent and communicate with the study team - Currently under the active care of a dietitian with a follow-up appointment scheduled	Patients in Australian sample (*n* = 32) M/F: 16 (50%)/16 (50%) Mean BMI: 26.1 (±7.0) Patients in Hong Kong sample (*n* = 42) M/F: 16 (38.1%)/26 (61.9%) Mean BMI: 19.7 (±3.5) 69% of patients have a caregiver, in the Australian site; 100% of patients in the Hong Kong site. Caregivers in Australian sample (*n* = 12) M/F: 5 (42%)/7 (58%) Relationship to patient: Spouse/partner 8 (67%), children 2 (17%), parents 1 (8%), other 1 (8%). Caregivers in Hong Kong sample (*n* = 42) M/F: 10 (23.8%)/32 (76.2%) Relationship to patient: Spouse/partner 21 (50%), children 13 (31%), parents 5 (11.9%), other 3 (7.1%).	Patients in Australian sample Gastrointestinal (25%), gynecological (9%), lung (6%), skin (13%), urological (38%), other (9%). Patients in Hong Kong sample Gastrointestinal (47.6%), gynecological (7.1%), lung (28.6%), urological (11.9%), other (4.8%).	Not declared.	Inadequate food intake and weight loss, which are associated with risk of malnutrition, frequently occurs among cancer patients. Those at advanced stages of cancer are particularly vulnerable to severe malnutrition due to complex pathophysiological factors including tumor-induced inflammatory responses and metabolic disorders. (Fearon, 2011).
Sim, 2022	- ≥ 20 years old - New diagnosis of gastrointestinal malignant tumor - Stages from II to IV - Receiving one or more cancer therapies without taking any nutritional supplements.	Patients who had acute infectious diseases, cardiac insufficiency, hepatic insufficiency, or patients receiving hemodialysis were excluded.	M/F: IG 13 (72.2%)/5 (27.8%) CG 19 (86.4%)/3 (13.6%) Average body weight loss: IG 6.01%, CG 5.06%	Diagnosis site CG:*n* = 1 esophagus, *n* = 1 duodenum, *n* = 5 stomach, *n* = 4 pancreas, *n* = 3 colon, *n* = 1 cecum, *n* = 3 rectum Cancer stage CG: stage II *n* = 2, stage III *n* = 3, stage IV *n* = 13 Diagnosis site IG:*n* = 1 esophagus, *n* = 3 gallbladder, *n* = 1 duodenum, *n* = 3 stomach, *n* = 8 colon Cancer stage IG: stage II *n* = 2, stage III *n* = 8, stage IV *n* = 12	Treatment CG: chemotherapy *n* = 10, surgery *n* = 1, chemo + surgery *n* = 6, chemo + radiation + surgery *n* = 1 Treatment IG: chemotherapy *n* = 16, chemo + radiation *n* = 1, chemo + surgery *n* = 5	Cancer cachexia is a multifactorial condition influencing 50%–80% of cancer patients and it is responsible for 20% of cancer deaths. (Warren, 1932; Fearon et al., 2011)
Bagheri, 2023	- Age ≥ 40 years with oral feeding - Diagnosis of colorectal cancer in stages 2–4 based on TNM UICC 2010 system (on the oncologist detection) - Presence of cachexia based on GLIM criteria - Patients' functional status - ≥ 70% according to Karnofsky scale	Presence of serious underlying diseases like renal and/or hepatic disorders and/or history of allergy to the Mediterranean diet components.	IG M/F: 15 (65.2%)/8 (34.8%) BMI: 23.36 ± 3.59 CG M/F: 17 (73.9%)/6 (26.1%) BMI: 22.63 ± 3.78	IG Cancer type: Rectum 19 (82.6%), colon 4 (17.4%) Cancer grade: G2 3 (13%), G3 18 (78.3%), G4 2 (8.7%) CG Cancer type: Rectum 19 (82.6%), colon 4 (17.4%) Cancer grade: G2 2 (8.7%), G3 18 (78.3%), G4 3 (13%)	Treatment CG: Chemotherapy *n* = 4 (17.4%), radiotherapy *n* = 19 (82.6%) Treatment IG: Chemotherapy *n* = 4 (17.4%), radiotherapy *n* = 19 (82.6%)	GLIM criteria were applied. The GLIM criteria included three phenotypic criteria (weight loss, low body mass index, and muscle mass loss) and two etiologic criteria (reduced food intake or reduced food absorption and the presence of inflammation or disease). To diagnose cachexia, there must be at least one phenotypic criterion and one etiologic criterion.
Buonaccorso, 2023	- Age > 18 years - Histologically confirmed cancer diagnosis - Presence of refractory cachexia and cachexia - Presence of the caregiver	- Presence of an important mental disorder or dementia - Severe sensory deficit - Presence of diffuse bone metastases that put the patient at risk of fracture during exercise	M/F: 15 (65.2%)/9 (37.5%) Mean BMI: 22.12 (±4.41) Cachexia: Reversible 20 (83.3%), refractory 4 (16.7%)	Pancreatic cancer 6 (25.0%) Lung cancer 5 (20.8%) Renal cancer 3 (12.5%) Upper GI cancer 3 (12.5%) Bladder cancer 2 (8.3%) Other 5 (20.8%)	Not described, but all patients were under active treatments.	Cachexia was assessed and measured by the guidelines of the European Society for Clinical Nutrition and Metabolism (ESPEN) guidelines and with the Malnutrition Universal Screening Tool (MUST). The stage of cachexia was defined by Fearon criteria.

M/F, male/female; PS, performance status; WL, weight loss; BMI, body mass index; CRP, C-reactive protein; GI, gastrointestinal; NSCLC, non-small cell lung cancer; ONS, oral nutritional supplement; MUAC, mid-upper arm circumference; BSC, best supportive care; AKPS, Australia-modified Karnofsky Performance Scale; PG-SGA, Patient-Generated Subjective Global Assessment; MUST, Malnutrition Universal Screening Tool; IG, intervention group; CG, control group.

First, ten out of fifteen authors cited Fearon's cachexia definition,^4^ with an increased incidence in the most recent articles. Consequently, the presence of cachexia was challenging to compare.

The authors defined different inclusion criteria related to the stage of the disease, varying from new diagnosis^24^^,^^29^ to the presence of advanced cancer.^22^^,^^31^^,^^33^^,^^34^ The criteria to define the presence of cachexia were also quite different. The authors included mainly patients with a weight loss > 5% in the last 6 months,^18^, ^19^, ^20^^,^^23^^,^^26^, ^27^, ^28^^,^^31^ according to Fearon's definition.^4^ Other characteristics used to describe the sample included the risk of malnutrition, which was generally high, and the Karnofsky Performance Status (KPS) scores. Despite the critical weight loss, the patients' BMI generally indicated a healthy weight (18.5–24.9 kg/m^2^).

The population displayed variation in terms of the site of the disease. Eight studies referred to gastrointestinal cancer (esophagus, gastroesophageal junction, gastric or colorectal cancer, biliary) and pancreatic cancer,^18^, ^19^, ^20^^,^^24^^,^^25^^,^^30^^,^^31^^,^^33^ and one to breast cancer.^28^

The presence of active treatments during the interventions was also highly variable between studies. Ten studies included the population under active treatment;^18^, ^19^, ^20^, ^21^, ^22^^,^^25^^,^^26^^,^^29^^,^^30^^,^^34^ one included patients waiting to start the first line of chemotherapy;^24^ two included patients who had to be out of any treatment plans (chemo-radiotherapy) during the period required for the intervention.^18^^,^^19^ Four authors did not state whether patients were under cancer treatment.^23^^,^^27^^,^^32^^,^^33^

Two studies included patients no longer receiving potentially curative treatment and patients under best supportive care.^22^^,^^25^

Regarding the variables reported, the only common parameters among the studies were gender, age, and BMI.

Only three studies planned the intervention on the dyad.^22^^,^^26^^,^^33^

All participants were outpatients.

### Non-pharmacological intervention

Four non-pharmacological interventions were employed: nutritional counseling, acupuncture, exercise, and psychoeducational/psychosocial (Table 4).

**Table 4:** The types of non-pharmacological interventions.

Author, year of publication	Intervention	Healthcare professional that provides the intervention	Comparison	Adherence	Dropout reasons	Results
Hopkinson, 2010	Support offered by MAWE-trained clinical nurse specialists. The components of MAWE are “breaking through the weight loss taboo”, “telling healing stories”, “managing conflict”, “support for eating well”, and “support for self-action”. These components are delivered during home consultations between nurse, patient, and caregiver. Delivery is supported by a pack of information leaflets entitled “Living with Changes in Eating”.	Nurse	Usual care	50/65 (77%) IG 25/35 (71%) CG 25/30 (83%)	Death (*n* = 7) A decline in clinical condition (*n* = 4) Discharge (*n* = 1) Another reason unrelated to the study (*n* = 3)	The intensity of eating- and weight-related distress was greater in the control group than that in the MAWE group. From the qualitative analysis: The thematic and content analysis found that MAWE was perceived as helpful by 1) supporting eating well with advanced cancer and 2) supporting self-management.
Faber, 2015	Dietary counseling +2 doses (2 × 200 mL sip feed) of active medical food for patients in the 0–5% WL group and at least 2 doses for patients in the ≥ 5% WL group. Active medical food is an energy-dense (163 kcal/100 mL), nutritionally complete oral supplement (FortiCare) that is high in protein and leucine (9.9 g protein/100 mL and 1.1 g free leucine/100 mL) and is enriched with emulsified fish oil (0.6 g EPA and 0.3 g DHA/100 mL), specific oligosaccharides (1.2 g galactooligosaccharides and 0.2 g fructooligosaccharides/100 mL) and a balanced mix of vitamins, minerals, and trace elements.	Not described	Dietary counseling +2 doses (2 × 200 mL sip feed) of the Control product daily for patients in the 0–5% WL group and at least 2 doses for patients in the ≥ 5% WL group. The Control product is for the 0–5% WL group, a non-caloric placebo product, and for the ≥ 5% WL group, an energy-dense (163 kcal/100 mL) iso-caloric standard nutritional product.	24/31 in the IG (77%). 23/33 in the CG (70%). Compliance (> 75% of the minimum amount of product): IG 89% and CG 87%	Start treatments 8/64, adverse event 2/64, withdrew consent 1/64, disease progression 1/64, other reasons 5/64	After 4 weeks, no differences regarding: - ConA-stimulated T-lymphocyte proliferation - Cytokine production in PBMC - White blood cells and number of NK-lymphocytes and NK-cell activity - Serum concentrations of inflammatory cytokines (IL-6, IL-1β) and CRP - Prealbumin and albumin concentration - QoL - Dysphagia score Significant increase in: - Body weight in the IG compared with the CG (*P* < 0.05) - ECOG PS improved in 17.4% of the patients in the IG and 0% in the CG, stable in 65.2% of the patients in the IG and 72.7% in the CG, and worsened in 17.4% of the patients in the IG and 27.3% in the CG. - Serum PGE2 levels in the CG (*P* = 0.01). - Total n-3 PUFAs, EPA, DPA and DHA (*P* ≤ 0.001) in the IG compared with the CG Significant decrease in - body weight in patients in CG ≥ 5% WL - Serum concentrations of PGE2 in the IG Total n-6 PUFAs, AA and the ratio n-6/n-3 PUFAs (*P* ≤ 0.001) in the IG compared with the CG.
Focan, 2015	Standard cachexia management + mindfulness and diet workshops (4 double workshops every 2 weeks) for a maximum of 10 patients conducted alternatively by psychologists and dieticians. In the diet workshops, foods had to be appraised through the five senses. Enrichment techniques and tasting of dishes at the level of the taste, the sense of smell, and the texture (touch) were developed.	Psychologist and dietitian	Standard cachexia management (standard dietetic support and eventual nutritional supplements according to estimated patient needs).	12/28 (43%) intervention group Adherence in the control group was not declared.	Not declared.	Significant increase of: - Body weight (+1.32 kg vs −1.47 kg; *P* < 0.01) - BMI (+0.31 vs −0.57, *P* = 0.04), - Clinical WHO/ECOG indices (57.1% vs 5.5%, *P* = 0.004) - Emotional function, fatigue and digestive disturbances (nausea, vomiting, constipation) (EORTC QLQ) - Faculty of observation (FFMQ) (*P* < 0.05). No differences between data at t1 and t2. No significant differences with regard to biological parameters, quantitative or relative qualitative calorie intakes, or nutritional indices.
Grundmann, 2015 and Yoon, 2015	Acupuncture treatment was administered by a qualified acupuncturist at his private practice. The intervention was provided to participants once prior to chemotherapy, once per week for 1 h between chemotherapy cycles, and once after chemotherapy. The total number of acupuncture treatments per participant was eight sessions during about eight weeks, which covered up to two chemotherapy cycles. The standards for reporting interventions in Clinical trials of acupuncture (STRICTA) recommendations were used as a guideline. All subjects received the same primary acupuncture points, including both auricular and body acupuncture points. Each participant received additional acupuncture interventions, which consisted of secondary acupuncture points, to address any specific symptoms that occurred to participants during the study and affected weight loss as well as appetite.	Acupuncturist	Not applicable	7/7 patients completed the intervention (100%)	Not applicable	*Results from BIA* Stability in: - Total body water (+1%) - Extracellular fluid (+2%) - Intracellular fluid (+1%) - Fat-free mass (+1%) Decrease in: - Body weight (−2.17 lbs (0.98 kg) (−1.3%)) - BMI (−1%, 0.37 ± 0.59) - Phase angle (−7%, −0.32 ± 0.52) - Fat mass (−6%) *Results from other outcome measures* Increase in: - Appetite (+3.04) - SNAQ scores (+4.14). - KPS score (+7.13 points, 6/7 participants maintained or improved their KPS score).
Kapoor, 2017	30 min of dietary counseling by a qualified nutritionist +100 g of IAtta, to be consumed every day in addition to their daily dietary intake for 6 months. Patients collected 14 packets of IAtta every fortnight during their appointments. Each 100 g of IAtta contained a mixture of roasted bengal gram flour, roasted barley flour, roasted soybean flour, flaxseed powder, and dried *Amaranthus spinosus* powder. The caregiver was advised to make unleavened flat breads *(chapatis).* On average 3 flat breads could be prepared from each pack, which provides approximately 400 kcal. Each 400 kcal consists of 50% daily protein requirement, 75% daily fat requirement, and 30%–50% of iron, calcium, and vitamin A.	Nutritionist	30 min of dietary counseling by a qualified nutritionist (twice a month). Patients were advised to increase the frequency of homemade meals and encouraged to consume energy- and protein-dense food products. Depending on the physical status of the patients, low levels of physical activity (walking and/or stairs) and participation in household activities were encouraged during counseling sessions.	33/63 patients (52%) completed the final follow-up at 6 months: 17/30 in the IG (57%) and 15/33 in the CG (45%).	Dropout causes were death (13 pts, four in CG and nine in IG) and loss to follow-up due to traveling difficulties, being bedridden or financial problems (18 pts, 14 in CG and four in IG). No demographic or clinical differences among the patients who dropped out compared with the ones who finished the study.	In the IG: - No significant body weight gain - Significant increase in body fat (*P* = 0.0029) - Significant increase in energy intake (*P* = 0.001) and macronutrient intake - No significant increase in PG-SGA score - Significant improvement in QoL, as fatigue (*P* = 0.002) and appetite loss (*P* = 0.006). - Maintaining of physical activity levels, from 33.6 ± 3.9 to 31.9 ± 2.7 METs (*P* = 0.274). - Significant improvement in pain (*P* = 0.012). In the CG: - Significant reduction in body weight (*P* = 0.003) and MUAC (*P* = 0.006). - Significant decrease in body fat (*P* = 0.032). - PG-SGA score did not change (from 9.4 ± 2.6 to 9.5 ± 2.4, *P* = 0.863). - Significant reduction in physical activity recall from 30.7 ± 2.7 to 28.0 ± 2.5 METs (*P* = 0.004). - Significant decrease in global health status (*P* = 0.018) and social functioning (*P* = 0.004). - Significant improvement in pain (*P* = 0.029).
Parmar, 2017	The multimodal interventions offered by the CNR-JGH clinic consisted of: - Dietary counseling and support, with the aim of achieving dietary intakes of at least 30 kcal/kg and 1.3 g protein/kg body weight. - Individualized exercise intervention plan, which included exercises to improve strength and endurance, performed up to twice a week at the hospital, supervised by the physiotherapists, or at home. - Symptom control through optimization of appropriate medication and, where appropriate, liaison with oncology and other medical, psychosocial, and community services. Patients attended the clinic at 6-week intervals, but this schedule was adjusted depending on the clinical need and patient availability.	Physician, nurse, physiotherapist and dietitian.	Not applicable	QoL scores were obtained from over 90% of patients at each clinic visit. Only 42% of the original cohort remained at visit 3. The proportion of dropouts between visit 1 and visit 3 was 50%–66%.	- Deterioration of conditions and need for end-of-life care - Rapid improvement of clinical condition - Unexpected medical deterioration - Poor motivation or other barriers to attending the clinic.	Consistent and statistically significant improvements in FAACT total (Visit1-3 +7.3), TOI (Visit1-3 +7.7), physical (Visit1-3 +2.3), and cachexia (Visit1-3 +4.1) subscales, over each visit interval, but not for the social and emotional subscale. Even though features such as being female, having GI cancer, high symptom scores, and poor performance status were associated with poorer QoL scores, the presence of these features at visit 1 did not preclude the possibility of improvements in QoL during the period of the clinical intervention. The greatest benefits of QoL were seen in those patients who gained weight and improved their 6 MWT.
Grundmann, 2019 and Yoon, 2019	Eight weekly acupuncture sessions of 45–50 min in consecutive weeks, provided by a certified acupuncturist. The selected 23 auricular and body acupuncture points (same for each patient) were linked to specific biological factors that affect the processes involved in the initiation, progression, or maintenance of cachexia (anti-inflammatory/immunomodulation, stress/autonomic nervous system, anorexia, and muscle wasting) using auricular acupuncture and traditional Chinese medicine. All the targeted points were needled with the patients fully dressed and in a supine position. Acupuncture needles were single-use, sterile stainless steel, and disposable, measuring 0.20 × 15 mm or 0.20 × 30 mm. The auricular points were located using their standard anatomical location and needled at proper needling depth (0.5–1.5 cm) bilaterally. Needles were retained for 15–20 min followed by the application of manual stimulation with an even rotating method until the needling (de qi) sensation or needle grasp sensation was obtained.	Acupuncturist	Eight weekly acupuncture sessions of 45–50 min in consecutive weeks, provided by a certified acupuncturist. Needles were applied to 5 acupuncture points that were not specific to the mechanisms of cachexia (headache, sore throat, or nasal congestion).	30 patients (79%) completed the 8-week intervention (attrition rate of 21%) IG: 15/20 (75%) CG: 15/18 (83%)	The reasons for withdrawal from the study after the first treatment were primarily related to extra transportation arrangements and decreased interest in the study. Of the two patients completing five treatments, one was dropped by the research team due to a protocol violation, while the other withdrew for personal reasons.	Increase in: - FFM (+1.671%, *P* = 0.016) in IG and CG - Leptin levels (+34% CG, +37% IG, > in males, non-significant). - Prealbumin levels in the CG - LDH activity in the CG, significant compared with the IG (*P* = 0.04). - Ratio of ECW to ICW (an unspecific indicator of inflammation and cellular integrity) in the IG compared with the CG (*P* = 0.05). - TNF-α levels in the IG (*P* = 0.04). Decrease in: - Ghrelin levels (−17%, not significant) in the IG Stability in: - FFM in the IG - Body weight in both groups - CRP levels in both groups Patients did not report any adverse events associated with the acupuncture. A decrease in leptin in the male intervention (MI) group corresponded to higher appetite and weight gain. The elevated ECW/ICW ratio indicated an inflammatory response in the MI group.
Yuliatun, 2019	Eight 30-min sessions of manual acupuncture were performed every two days by a certified acupuncturist. Acupuncture needles were single-use, sterile stainless steel, and disposable, a brand of Huan Qiu made in China measuring 0.25 × 25 mm for acupuncture points Hegu (LI-4), Zusanli (ST36), Sanyinjiao (Sp6), Xuehai (Sp10), Neiguan (P6) and Dazhui (GV14), and needle size 0.13 × 20 mm for acupuncture point of dicang (ST4). Acupuncture points were located using standard anatomical location and needle at proper needling depth (0.5–1.5 cm) bilaterally.	Acupuncturist	Not applicable	7/7 patients completed the intervention (100%)	Not applicable	Body weight, BMI, and FFM did not significantly change during the study. 11.63% of patients had a body weight decrease at the end of the intervention. 19.4% of patients had a BMI decrease at the end of the intervention.
Kamel, 2020	Progressive resistance training, achieved through 60-min machine-based exercise sessions carried out twice a week for 12 weeks. Groups of one to four patients underwent the sessions at a time, supervised by specialized physical therapists. At each session, general flexibility exercises and one set for the first exercise of upper and lower extremities were performed before the training program at a lower training intensity, to ensure adequate warm-up. Machine-based resistance exercises followed the warm-up. Following two familiarization sessions, one to two sets of the first five exercises with 20 repetitions were performed by participants for a four-week adjustment phase of low to moderate intensity (50%–60% 1-RM). Beginning at week 5, the number of exercises was increased to eight per session; the patients were asked to perform three sets with 8–12 repetitions, with a moderate to high frequency (60%–80% 1-RM). In case of clinical complications (e.g., infections, fever, high or low blood pressure, etc.), resistance training was stopped immediately. Participation in the study could be interrupted at any moment by the oncologist or the patient him/herself.	Physiotherapist	Nutritional and psychosocial support, no exercise regimen. The physiotherapist contacted the patients once a month by phone to inquire about the possible negative outcomes of cancer therapy.	33/40 patients completed the study (83%): 17/20 (85%) in the IG and 16/20 (80%) in the CG.	Dropout causes were death (*n* = 2; 1 IG,1 CG), withdrawal (*n* = 3; 2 IG, 1 CG) and disease progression (*n* = 2; 2 CG).	Significant increase in: - Walking efficiency in the 400 m walk test (*P* = 0.005) - 6-m usual MWT (*P* = 0.001) - Chair rise test (*P* = 0.001) - Peak torque of knee extensors (*P* = 0.004) - Elbow flexors (*P* = 0.001) and elbow extensors (*P* = 0.001) - Maximum voluntary isometric contraction of the knee and elbow flexors and extensors (*P* < 0.01) - Lean mass of the upper limb, lower limb, and appendicular skeletal muscles (*P* < 0.001). No significant difference in: - Body fat percentage - 6-m fast MWT
Latenstein, 2020	Dietetic consultation	Dietitian	Not applicable	Not applicable	Not applicable	Patients who underwent surgery − 63% had cachexia at baseline, > 53% had dietetic consultation − 66% had insufficient protein score, > 37% had dietetic consultation Patients who received palliative chemotherapy − 77% had cachexia at baseline, > 52% had dietetic consultation − 76% had insufficient protein score, > 32% had dietetic consultation Patients with best supportive care − 82% had cachexia at baseline, > 71% had dietetic consultation There were no differences in overall survival, but it was shorter in patients with more severe weight loss (≥ 10%).
Bland, 2021	When patients with cancer presented involuntary weight loss, anorexia symptoms, and/or functional declines, they were referred to the clinical service and screened and triaged over the phone by a nurse practitioner. At the first visit, patients and caregivers met with the entire multidisciplinary care team for 80 min. The care team included a palliative medicine physician, nurse practitioner, dietitian, and physiotherapist. With shared decision-making, the multidisciplinary care team formulated an individually tailored multimodal treatment plan, which could include drugs for symptoms, nutritional counseling (diet advice and oral nutritional supplements), and physiotherapy advice (home-based exercise). Follow-up visits lasted approximately 40 min and occurred every four to six weeks.	Palliative medicine physician, nurse practitioner, dietitian, and physiotherapist	Not applicable.	Not applicable.	101 (62%) patients died at follow-up (not specified if visit 2 or 3).	No significant main change in body weight (*P* = 0.907), hand-grip strength (*P* = 0.734), and 30s sit-to-stand (*P* = 0.133) occurred over time. Significant improvements in overall QoL (*P* < 0.001), physical function (*P* < 0.003), and emotional function (*P* < 0.001) were detected between the first and second visits. Results were maintained at the third visit. Significant main effects were also found for all EORTC QLQ-C15-PAL symptoms, except for constipation (*P* = 0.078), insomnia, and breathlessness. FAACT outcomes improved between the first and second visit (FAACT total score *P* < 0.001, the FACT-G total score *P* < 0.001, the TOI *P* < 0.001, anorexia-cachexia symptoms *P* < 0.001, physical *P* < 0.001, emotional *P* = 0.005, and functional wellbeing *P* = 0.001), and they remained stable after the third visit.
Molassiotis, 2021	The intervention provided three structured sessions (2–3 h) of dietitian direct contact time over a 4-week period, inclusive of telehealth (Australian site only) or telephone follow-ups to monitor, reinforce and adjust goals. The context of the intervention was around nutrition impact symptoms, quality of life and food or eating-related psychosocial concerns in patients and caregivers through nutrition counselling, as well as addressing nutrition-related communication between the dyads, rather than solely achieving sufficient energy/protein intake, which is a common approach in traditional dietary interventions. The intervention also included a culturally adapted booklet that was provided to the patients and their caregivers. If patients were admitted to the hospital during the intervention period, the ward dietitian provided dietetic care to the patient while they were an inpatient, and the research dietitian continued with the intervention following discharge.	Dietitian	Subjects in the control group received the usual care, which may have involved some nutrition advice and symptom management. More specifically, in Hong Kong usual care involved nutritional advice and symptom management by the palliative care team in the hospitals. Referral to a dietitian was offered when medically indicated by physicians. An assessment was usually conducted every 4–6 weeks, depending on whether the patient achieved improvements in dietary intake. Following completion of outcome assessments, patients were given the option to participate in intensive dietitian-delivered nutrition counseling off the trial.	Recruitment feasibility 22/34 (64.7%) patients in the IG group completed the intervention vs 30/40 (75%) in the CG. 17 patients in the IG group completed the week 5 assessment vs 21 patients in the CG.	Dropout reasons were withdrawal (*n* = 5), unable to complete within the study timeframe (*n* = 1), re-hospitalized (*n* = 3), passed away *n* = 10, no mood (*n* = 3).	Acceptability of assessment tools The assessment tools used were generally acceptable, with a rating ≥ 5.18 (on a 0–10 point scale) in both sites. Outcome assessments Results showed a tendency for improvements in all patient outcome measures in IG compared with CG. These changes reached statistical significance (*P* < 0.05) for Eating-related distress and FAACT QoL, in the Australian sample, and Eating-related enjoyment, in the Hong Kong sample. Caregiver outcome measures showed a smaller and not statistically significant difference in all variables between IG and CG. Weight was maintained in the IG and decreased slightly in the CG. Clinically significant improvements were observed in the IG in terms of mean energy intake and mean protein intake.
Sim, 2022	The experimental group received regular nutrition counseling and education. Patients in IG were asked to take ONS twice a day (400 mL, 400 kcal). Patients were asked to record the amounts of ONS consumed, and weekly telephone counseling was used to determine and maintain compliance. ONS was a product enriched with omega-3 fatty acids (70 mg/200 mL) and arginine (250 mg/200 mL).	Dietitian	In addition to nutritional counseling and education, patients in the control group also received a weekly telephone call from a trained dietitian for further nutrition counseling.	40 patients (69%) concluded the study and were included in the final analyses (22 patients in the IG [55%], 18 patients [67%] in the CG).	nine patients dropped out in the IG (*n* = 3 death, *n* = 1 transfer, *n* = 1 no specific reason, *n* = 4 included in other studies). nine patients dropped out in the CG (*n* = 2 death, *n* = 2 transfer, *n* = 2 nausea, *n* = 1 poor condition, *n* = 2 no specific reason).	Nutritional status – PG-SGA scores The paired *t test* between week 0 and week 8 showed improvements in both groups; only in the IG, the results were statistically significant (*P* = 0.001). No differences were detected between IG and CG (*P* = 0.118). Quality of life score - EORTC-QLQ C30 The global health status score was increased only in the IG, but no differences were detected between IG and CG. Among functional scales, the role function score was significantly decreased in the CG (*P* = 0.001) at week 8, while that of the IG did not change. Fatigue, nausea and vomiting, and all other symptom scales showed a steady decrease in the IG and a steady increase in the CG, between baseline and week 8. Biochemical markers No significant difference between groups was observed, and the concentration of inflammatory cytokines also did not exhibit any differences.
Bagheri, 2023	All patients were visited by expert dietitians, who prescribed a Mediterranean diet regime with extra virgin olive oil (EVOO) only for the IG group. For the Mediterranean diet group, the required energy for the patients was estimated according to the ASPEN guidelines, which started with 25 kcal/kg body weight per day and then reached 35 kcal/kg body weight per day within two weeks. The weekly dietary menu was designed according to the Mediterranean regimen, and EVOO was provided free of charge to the patients during the intervention period. Moreover, for the convenience of the patients and to increase their adherence to the protocol, the diet was personalized based on their tastes. The duration of the intervention was eight weeks.	Dietitian	All patients were visited by expert dietitians. In the control group, routine According to the clinical guidelines, nutritional recommendations regarding weight gain and prevention of weight loss in cancer patients were given as brochures.	40/46 (86.9%) 46 patients entered the randomization stage, and 40 patients completed the study.	In the IG: *N* = 2 did not complete the study *N* = 1 did not follow the diet. In the CG: *N* = 2 did not complete the study *N* = 1 died.	Anthropometric indices Average weight (changes from baseline in IG 0.94 vs −1.81, *P* = < 0.001), lean body mass (IG vs CG: 0.35 vs −1.35, *P* = 0.01), fat mass (IG vs CG: 0.76 vs −0.58, *P* = 0.002), fat percentage (IG vs CG: 0.98 vs −0.78, *P* = 0.02) and muscle strength (IG vs CG: 1.56 vs −2.29, *P* < 0.001) significantly increased in IG. Quality of life PG-SGA score decreased significantly (IG vs CG: −4.04 vs 1.86, *P* < 0.001), showing the improved nutritional status in the Mediterranean diet group. In IG the score for global health status (IG vs CG: 6.78 vs −3.95, *P* = 0.02), physical performance score (IG vs CG: 8.44 vs −6.32, *P* < 0.001), appetite (IG vs CG: −6.67 vs 13.29, *P* = 0.01), and diarrhea (IG vs CG: −2.14 vs 15.48, *P* = 0.02) were improved significantly. Inflammatory markers The mean serum level of TNF-α was significantly decreased in the Mediterranean diet group (IG vs CG groups: −0.47 vs 0.35, *P* < 0.001). The mean serum levels of hs-CRP (IG vs CGs: −63.23 vs 1,406.62, *P* = 0.01) and IL-6 (IG vs CG: −0.02 vs 1.09, *P* < 0.001) were significantly increased in the control group. The average of total serum protein in the CG was significantly decreased compared to IG (IG vs CG: 0.04 vs −0.46, *P* = 0.008).
Buonaccorso, 2023	The intervention included psycho-educational and rehabilitative interventions in addition to standard care. The psycho-educational component of the intervention included three weekly meetings for dyads led by three trained nurses. The face-to-face consultations aimed to help the dyad cope with involuntary weight loss and declining appetite by seeking to strengthen individual and dyadic coping resources. The dyads were given an information booklet, which included a description of cancer cachexia and the major emotional reactions of patients and families. The rehabilitative component of the intervention was conducted by two trained physiotherapists. It included three individual outpatient sessions in two months and three home sessions of exercises per week, carried out by the patient on his or her own or with the help of the caregiver, for a total of at least 24 home sessions +3 outpatient face-to-face meetings with the physiotherapists over eight weeks.	Nurse and physiotherapist	The standard care was a specialized PC visit.	Twenty-four dyads were evaluated at baseline (T0), sixteen (66.6%) at T1, 11 (45.8%) at T2, and six (25%) at T3 final follow-up. 20/24 dyads completed the psychoeducational component, which was feasible for 83.3% of the sample. 6/24 dyads completed the rehabilitative component, which was feasible for 25% of the sample.	18/24 patients withdrew from the study: - Death (*n* = 1) - Deterioration of clinical conditions (*n* = 16) - Difficulties coming back for follow-up (*n* = 11). One patient was affected by a pathological fracture, but this did not occur during exercise. 12/24 patients (50%) died within three months of enrollment.	QoL decreased over time, and the caregiver burden diminished between enrollment and T2. Upper limb strength was substantially stable in the first month (between T0 and T2) and worsened at T3. Lower limb physical performance measured by the 30-s sit-to-stand test showed better scores at T3. Considering the scores of patients evaluated at two months follow-up (T3), the trend showed no deterioration in QoL, caregiver burden, or patients' physical performance. Qualitative data from six interviews showed a good level of acceptability of the bimodal intervention.

MAWE, Macmillan Approach to Weight and Eating; IG, intervention group; CG, control group; WL, weight loss; EPA, eicosatetraenoic acid; DHA, docosahexaenoic acid; PBMC, blood peripheral mononuclear cells; QoL, Quality of Life; PS, performance status; BMI,: body mass index; BIA, bioelectrical impedance analysis; SNAQ, Simplified Nutritional Appetite Questionnaire; KPS, Karnofsky Performance Scale; PG-SGA, Patient-Generated Subjective Global Assessment; MET, metabolic equivalent unit; MUAC, mid-upper arm circumference; FAACT scale, Functional Assessment of Anorexia/Cachexia Treatment Scale; TOI, Trial Outcome Index; MWT, minute walking test; FFM, fat-free mass; ECW, extracellular water; ICW, intracellular water; RM, repetition maximum; EORTC QLQ-C15-PAL, EORTC Quality of Life Questionnaire Core 15 Palliative Care; ONS, oral nutritional supplement.

Six interventions were multimodal (35.3%).^23^^,^^26^^,^^31^, ^32^, ^33^, ^34^ In three studies, the non-pharmacological intervention was the control arm of the randomized controlled trial (RCT).^18^^,^^20^^,^^25^ Latenstein aimed to assess dietetic consultation alone for patients with pancreatic cancer and its effect on survival and patient-reported outcome measures (PROMs).^25^ Acupuncture was always studied as a single intervention^18^^,^^19^^,^^28^ or compared with a placebo.^20^

Nutritional counseling, or dietetic consultation, was conducted by a trained dietician or nutritionist, who advised increasing the consumption of energy-dense and high-protein foods and the overall dietary intake.^23^^,^^24^^,^^27^^,^^29^^,^^30^^,^^33^^,^^34^ In Faber's and Sim's studies, dietary counseling was the control arm of a pharmacological randomized controlled trial.^24^^,^^29^ In Bagheri et al., the nutritional counseling focused on prescribing a Mediterranean diet, with specific amounts of kcal/kg and olive oil consumed; the regimen was based on a varied diet and the patient's taste.^30^

The authors generally did not provide much detail on the frequency and content of visits with the dietician. Only Latenstein and colleagues explained the objectives of the visits with end-of-life patients in detail; the focus of the intervention was on the needs of the patients, aiming to improve comfort and support quality of life.^25^

Psychoeducational/psychosocial interventions aimed to reduce the emotional burden associated with cancer cachexia by empowering patients and families to cope with the dysfunctions and derangements of cachexia, thus improving quality of life.^6^ Psychoeducational/psychosocial support comprised two distinct types of interventions: (1) meetings on the meaning of food, experiences, and concerns regarding eating-related distress, the impact of weight loss on the patient regarding self-image and self-esteem, and on the dyad^22^^,^^26^^,^^33^ in relation to interactions about mealtime and coping strategies; (2) mindfulness workshops on the theme of taste through the five senses.^23^ The interventions were conducted by different healthcare professionals, including nurses,^22^^,^^26^ psychologists,^23^ and dieticians.^27^^,^^33^

In Kapoor's study, the same nutritionist who delivered the dietary counseling added advice to increase the low levels of daily physical activity.^27^ In the studies by Kamel^31^ and Parmar,^34^ on the other hand, the rehabilitation intervention was more structured and was conducted by an experienced physiotherapist, with exercises to maintain strength, endurance, and flexibility.

The acupuncture intervention in the three studies differed in terms of the frequency of the sessions, varying from eight weekly sessions^18^^,^^19^ to eight sessions, one every two days.^28^ The acupuncturists applied needles to points specific to the mechanisms of cachexia.

The duration of the non-pharmacological interventions was variable. The acupuncture intervention ranged from 2.5 weeks^18^ to 18 weeks.^28^ The most extensive intervention was conducted by Kapoor,^27^ where dietary counseling was offered twice a month for 6 months. The duration of the interventions does not seem to be correlated to either the type of intervention proposed or the adherence achieved.

Based on the type of intervention, acupuncture achieved the best adherence, with 100% of patients completing the intervention during the pilot study^18^^,^^19^ and 75% in the RCT.^20^^,^^21^ Exercise alone had 85% adherence,^31^ whereas when combined with dietary counseling, the adherence decreased to 57%.^27^ Psychoeducational intervention alone reached 71% adherence in Hopkinson et al.^22^ and 83.3% in Buonaccorso et al.,^26^ while adherence to dietary counseling varied from 55% to 10%^24^^,^^29^ to 86.9%.^30^ When the three types of intervention were combined, adherence was 43%–65%.^23^^,^^33^ In the two retrospective studies, only 42% and 48% of patients, respectively, continued to participate in the intervention after 18 weeks^34^ and 12 weeks.^32^

The main reasons for dropout were death and a decline in clinical condition due to disease progression or re-hospitalization. The third reason was the withdrawal of consent due to decreased interest or personal reasons. Other reasons included financial problems, transportation arrangements, violation of protocol, or inability to complete within the study timeframe.

### Outcomes

The most common primary outcomes were body weight, BMI, and body composition (bioelectrical impedance analysis [BIA] measurements),^18^^,^^27^ followed by nutritional measures (food anamnesis, dietary intake, appetite, nutritional questionnaires),^24^^,^^25^^,^^27^^,^^30^ physical performance [KPS], physical activity, mobility),^27^^,^^31^^,^^34^ biomarkers,^20^^,^^24^^,^^27^^,^^28^^,^^30^ and psychological suffering assessed by multidimensional scales such as Functional Assessment of Anorexia Cachexia Therapy (FAACT) and European Organization for Research and Treatment of Cancer Quality of Life Questionnaire (EORTC QLQ)-C30, or a specific test for anxiety and depression (Hospital Anxiety and Depression Scale [HADS]). For caregivers, Molassiotis^33^ chose HADS and Buonaccorso used the Zarit Burden Scale.^26^ Hopkinson^22^ and Molassiotis^33^ chose the visual analog scale (VAS) to measure eating- and weight-related distress. Latenstein's primary outcome was overall survival.^25^ Nine out of 17 articles also include quality of life questionnaires (EORTC QLQ-C30, EORTC QLQ-C15-PAL, and FAACT scale).^23^^,^^26^^,^^27^^,^^29^^,^^30^^,^^32^, ^33^, ^34^

### Risk of bias in the included studies

The review aimed to describe non-pharmacological interventions for cancer cachexia reported in the literature rather than to assess their efficacy. Thus, a risk of bias assessment of the included studies was performed to provide additional information on the current literature about this topic rather than to assess the reliability of estimates coming from the studies.

The overall risk of bias was high for both randomized and non-randomized studies. We found only one randomized controlled trial with an overall judgment of “low risk” and three with an overall judgment of “some concerns” of risk of bias, while all the other studies were judged to be at “high risk of bias.”

The main drivers of risk of bias in the included studies were related to missing outcome data and measurement of outcomes among randomized studies and to bias in sample selection and inadequate follow-up rates among non-randomized studies.

The risk of bias graph and risk of bias summary are presented in Supplementary Figs. 1–4 by study design (randomized and non-randomized studies). Detailed judgments are reported in Supplementary Table 1 for randomized studies and in Supplementary Table 2 for non-randomized studies.

## Discussion

Of the 9308 titles screened, we included 17 articles corresponding to 15 studies. This review aimed to identify which non-pharmacological interventions have been studied for cancer patients with cachexia and refractory cachexia and are most often encountered in palliative care contexts.

We report some specific points that emerged from our data, and we think that focusing on these issues should help healthcare professionals to construct personalized interventions, particularly for advanced cancer patients.

### Multimodal component of the interventions and heterogeneity of the population

Only six interventions out of 15 were multimodal (39%),^23^^,^^26^^,^^31^, ^32^, ^33^, ^34^ even though the literature reported that a personalized treatment and multidisciplinary approach to evaluate the objective signs and relieve the symptoms is required.^2^ The most common multimodal components were nutritional counseling, exercise, and psychoeducation/psychosocial interventions, suggesting that a comprehensive and multidisciplinary approach could be necessary for this condition.^9^^,^^35^^,^^36^ A multimodal approach has strong theoretical backing but can be challenging to implement in clinical practice due to time and resource restraints.^9^

The studies analyzed included patients with different cachexia stages and cancer sites. Five out of 15 studies involved gastroenteric cancer patients. In 8 out of 15 studies, patients had ongoing active treatment. Although there has been greater adherence to the shared definition of Fearon and colleagues in recent years,^4^ these differences in population prevent making solid conclusions.^36^

The data showed heterogeneity in the duration of the interventions. Moreover, the long follow-up period of some studies raises doubts about the possible feasibility in clinical practice and on patients' adherence to such complex and multidimensional interventions.

Acupuncture has been studied as a single intervention. It seems to reveal a cultural approach, as it has been used primarily in Eastern countries,^18^^,^^19^ although the selected studies cover five continents (Europe, America, Asia, Africa, and Australia).

In the studies included in our review, the main reasons for dropout were death and a decline in clinical condition due to disease progression or re-hospitalization. As healthcare professionals, our focus should move from end-stage wasting to supporting patients’ nutritional and functional state early on, and needs-centered interventions would be desirable.^37^

Due to the heterogeneity of these elements, we could not proceed with a meta-analysis. However, this also makes it difficult to make a comparison or synthesis of what may be effective intervention elements for the management of cachexia. Further studies that consider these methodological challenges are needed.

### Outcomes

A particular reflection should be dedicated to the choice of outcomes. The most common primary outcomes were body weight and body composition, biomarkers, psychological questionnaires, and muscular strength. Nearly half of the studies also included quality of life questionnaires among the outcomes, with an increase seen in more recent studies. This is particularly important in palliative care settings, where the intervention should aim to improve the patient's quality of life, not only his/her weight or laboratory tests.^35^^,^^38^^,^^39^

### Interventions targeting the dyad: a future perspective

Our data shows that only three studies were dedicated to the dyad.^22^^,^^26^^,^^33^ As the literature suggests, the entire family system will be affected by and respond to the loved one with cancer cachexia.^37^^,^^40^^,^^41^ For example, patients who live with their partners report more eating-related distress than those who live alone.^40^ An extensive survey (76% response rate) of 702 bereaved family members of cancer patients in Japan showed that those who believed they forced the patient to eat to avoid death and those who believed they did not have correct information about cancer cachexia showed a higher risk of bereavement depression.^41^

### Implications for nursing practice

In the included studies, the healthcare professionals who conducted the interventions varied widely (nurses, dieticians, physiotherapists, psychologists), sometimes stepping outside their expertise. In Kapoor's study, for example, the same nutritionist who delivered the dietary counseling conducted the physical activity component, adding advice to increase the low levels of daily physical activity.^27^ The literature, on the other hand, suggests a coordinated intervention by a registered dietician, physiotherapist, palliative care nurse, psychologist, and palliative care specialist.^9^^,^^35^

A nurse should therefore be a member of this multidisciplinary team, which is composed of healthcare professionals with complementary areas of expertise. Though the contribution of some team members is clearly defined (e.g., the physiotherapist supports physical activity/exercise), the nurse's role has not been clearly differentiated.^13^ In Buonaccorso et al., nurses and physiotherapists attended a brief course together on the psychoeducational needs of patients and their caregivers in the context of cancer cachexia.^26^ In nursing education on the management of cancer cachexia, as well as for all other professionals, it is fundamental to tailor the training according to the cachexia stage, symptoms, emotional response, and social circumstance.^13^

### Limitations

This scoping review analyzed data from all over the world, which could give a global vision of the problem. Only peer-reviewed studies were included. Literature via other sources, such as clinical trial registers or pre-print databases, was not searched, so non-pharmacological interventions described in grey literature may have been missed. The high overall risk of bias in the included studies was mainly attributable to outcome measures and missing data due to loss at follow-up. Although the use of self-reported outcomes (e.g., quality of life, patient experience, and so on) is a crucial point for a patient-centered approach, it may introduce bias in assessing the effectiveness of interventions, especially in unblinded studies. At the same time, a high proportion of loss at follow-up is to be expected in trials of late-stage palliative care interventions and may introduce bias if not managed. Considering these points from the beginning of the study design makes it possible to implement strategies related to the choice of outcomes, assessment time points, and sample size, as suggested by the MORECare statements.^42^ Finally, studies before 2012 were not included because we noted an exponential increase in publications on this issue more recently. This could be considered a limitation in a scoping review, even though limiting the search to a specific period is quite common.

## Conclusions

This review clarifies which recent studies have been conducted to manage cancer cachexia with non-pharmacological interventions. The results show heterogeneous approaches with good patient adherence, sometimes combined in multimodal interventions. The studies have various methodological limitations, which make the results difficult to compare and apply. However, they are worthy of further research. Well-designed studies with a clear definition of cancer cachexia involving a homogenous population by type of cancer and active or non-active treatment are needed.^38^^,^^39^^,^^43^

To apply the interventions to patients in palliative care, it is essential to pay attention to the choice of outcomes, which should align with the patient's actual needs. Quality of life includes weight or laboratory tests, the management of mealtime, relationships with food and tastes, and support of psychological suffering. Caregiver involvement can be crucial when approaching the subject of food and its related experiences, so interventions involving the dyad will be central.^2^^,^^41^

## Ethics statement

Not required.

## Funding

This study was also partially supported by the Italian Ministry of Health-Ricerca Corrente Annual program 2025.

## CRediT authorship contribution statement

**EB**, **LB**, and **ST** were responsible for the planning, design, conduct, and reporting of the work. **EB** and **FF** performed the study selection and data extraction. **EB** and **FF** were involved in the study appraisal process. **EB**, **LB**, **FV**, and **ST** were involved in the data analysis and synthesis process, **MCB** was involved in the data extraction. All authors had full access to all the data in the study, and the corresponding author had final responsibility for the decision to submit for publication. The corresponding author attests that all listed authors meet authorship criteria and that no others meeting the criteria have been omitted.

## Declaration of competing interest

The authors declare no conflict of interest.

## Declaration of Generative AI and AI-assisted technologies in the writing process

No AI tools/services were used during the preparation of this work.

## Data availability statement

The authors confirm that the data supporting the findings of this study are available within the article.
