# Supplementary material for: Cancer cachexia: A scoping review on non-pharmacological interventions
Source: Asia Pac J Oncol Nurs. 2024 Mar 12;11(5):100438. doi: 10.1016/j.apjon.2024.100438 (PMC11107192; doi:10.1016/j.apjon.2024.100438)
Supplement: Multimedia component 1 [file mmc1.docx]

***
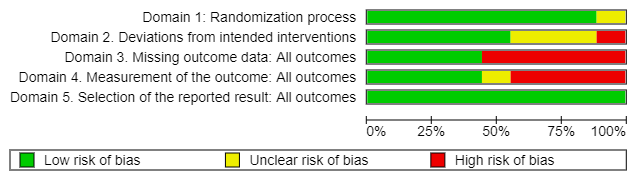
***

***Figure 1.*** Risk of bias graph: review authors' judgements about each risk of bias item presented as percentages across all included studies. (Randomized studies, RoB 2.0)

***
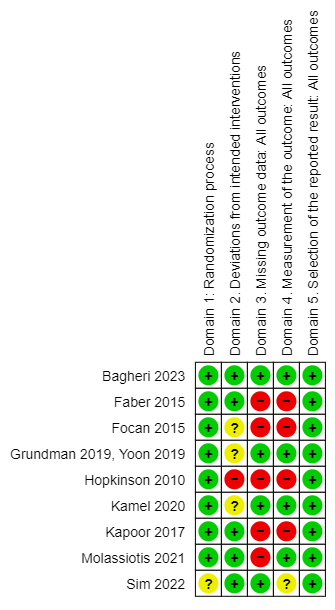
***

***Figure 2.*** *Risk of bias summary: review authors' judgements about each risk of bias item for each included study. (Randomized studies, RoB 2.0)*

***
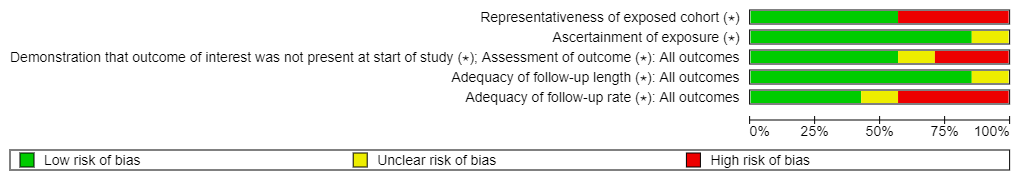
***

***Figure 3.*** *Risk of bias graph: review authors' judgements about each risk of bias item presented as percentages across all included studies. (Non-Randomized studies, Newcastle-Ottawa Scale (NOS)). Since only single-arm trials were retrieved and included in the review, three items of the NOS-Cohort studies (Selection of the non-exposed cohort, Comparability of cohorts on the basis of the design or analysis) were deemed “Not Applicable” and excluded by the assessment.*

***
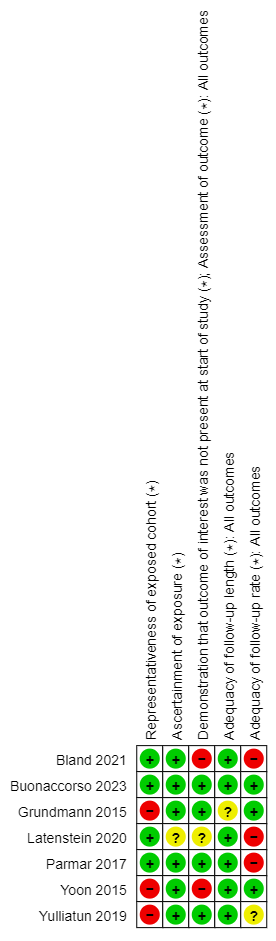
***

***Figure 4.*** *Risk of bias summary: review authors' judgements about each risk of bias item for each included study. (Non-Randomized studies, Newcastle-Ottawa Scale) Since only single-arm trials were retrieved and included in the review, three items of the NOS-Cohort studies (Selection of the non-exposed cohort, Comparability of cohorts on the basis of the design or analysis) were deemed “Not Applicable” and excluded by the assessment.*
