# Supplementary material for: Cancer cachexia: A scoping review on non-pharmacological interventions
Source: Asia Pac J Oncol Nurs. 2024 Mar 12;11(5):100438. doi: 10.1016/j.apjon.2024.100438 (PMC11107192; doi:10.1016/j.apjon.2024.100438)
Supplement: Multimedia component 2 [file mmc2.docx]

**Appendix – Table X. Risk of bias assessment of non-randomized studies.** For included non-randomized studies, risk of bias was assessed using the Newcastle-Ottawa Scale [ref]. For each item of the scale, the studies were judged by the authors to be at low, unclear or high risk of bias. The star foreseen by the Newcastle-Ottawa Scale scoring methodology (⋆) was assigned only to low risk of bias items. Since only single-arm trials were retrieved and included in the review, three items of the NOS-Cohort studies (Selection of the non-exposed cohort, Comparability of cohorts on the basis of the design or analysis) were deemed “Not Applicable” and excluded by the assessment.

**Bland, 2021**

**Risk of bias table**

| **Bias** | **Authors' judgement** | **Support for judgement** |
| --- | --- | --- |
| Representativeness of exposed cohort (⋆) | Low risk (⋆) | A total of 462 pts were screened and triaged to attend the service, of which 316 were able to attend at least once. |
| Selection of non-exposed cohort (⋆) | N.A. |  |
| Ascertainment of exposure (⋆) | Low risk (⋆) | The multidisciplinary visits are well described, both the first and the follow-up visits. Retrospective observational review  using routine data collected from patients attending  the Barwon Health Cachexia and Nutritional Support  Service in Geelong, Victoria, Australia |
| Demonstration that outcome of interest was not present at start of study (⋆) | Low risk (⋆) | Self-reported quality of life outcomes were collected, and they were different from the beginning to the end of the study.  Sit to stand test and Hand grip strand were also performed. |
| Specify how comparability is assessed - main confounder: T-stage (⋆) | N.A. |  |
| Specify how comparability is assessed - other confounders: site (⋆) | N.A. |  |
| Assessment of outcome (⋆) | High risk (⋆) | EORTCQOL and FACT outcomes were self-reported. |
| Adequacy of follow-up length (⋆) | Low risk (⋆) | 3 months follow-up |
| Adequacy of follow-up rate (⋆) | High Risk | 51% attended all 3 planned visits |

**Grundmann, 2015**

**Risk of bias table**

| **Bias** | **Authors' judgement** | **Support for judgement** |
| --- | --- | --- |
| Representativeness of exposed cohort (⋆) | High risk | Limited number of recruited patients, all with colorectal and/or gastrointestinal cancers undergoing chemotherapy and recruited by the cancer care coordinator and the attending oncologist. Not explained whether the recruitment was consecutive. |
| Selection of non-exposed cohort (⋆) | N.A. | Single-group design |
| Ascertainment of exposure (⋆) | Low risk (⋆) | Prospective study. Acupuncture treatments were administered by a qualified acupuncturist at his private practice. Intervention well described in the methods. |
| Demonstration that outcome of interest was not present at start of study (⋆) | Low risk (⋆) | All patients were evaluated before the start of the acupuncture intervention to establish a baseline. Main outcome: The BIA measurements for each time point were standardized to baseline measures and calculated as percentage change. |
| Specify how comparability is assessed - main confounder: N.A. (⋆) | N.A. | Single-group design |
| Specify how comparability is assessed - other confounders: N.A. (⋆) | N.A. | Single-group design |
| Assessment of outcome (⋆) | Low risk (⋆) | BIA was measured every other visit (total of 4–5 times) at the acupuncture physician’s office. All derived measurements were based on equations for healthy volunteer populations, which is a limiting factor for accurate determination of BIA measurements but has been commonly employed. |
| Adequacy of follow-up length (⋆) | Unclear | Unclear the distance between time points of BIA measurements for each patient. |
| Adequacy of follow-up rate (⋆) | Low risk (⋆) | No subject withdrew from the study. |

**Latenstein, 2020**

**Risk of bias table**

| **Bias** | **Authors' judgement** | **Support for judgement** |
| --- | --- | --- |
| Representativeness of exposed cohort (⋆) | Low risk (⋆) | All patients with pancreatic and periampullary cancer between 2015 and 2018 |
| Selection of non-exposed cohort (⋆) | N.A. |  |
| Ascertainment of exposure (⋆) | Unclear risk | The aim of the study was assessing the prevalence of cachexia, dietitian consultation and overall survival |
| Demonstration that outcome of interest was not present at start of study (⋆) | Unclear risk | Description of a population in a single time point |
| Specify how comparability is assessed - main confounder: T-stage (⋆) | N.A. |  |
| Specify how comparability is assessed - other confounders: site (⋆) | N.A. |  |
| Assessment of outcome (⋆) | Low Risk (⋆) | Percentage of cachexia, dietetic consultants and survival were collected in a specific population |
| Adequacy of follow-up length (⋆) | Low Risk (⋆) | There is 3 months follow-up |
| Adequacy of follow-up rate (⋆) | High risk | There is 3 months follow-up only for 53 pts out of 202 |

**Parmar, 2017**

**Risk of bias table**

| **Bias** | **Authors' judgement** | **Support for judgement** |
| --- | --- | --- |
| Representativeness of exposed cohort (⋆) | Low Risk (⋆) | Retrospective analysis of 405s patients who attended at least 1 clinic visit during the study period. 374 completed all the scales |
| Selection of non-exposed cohort (⋆) | N.A. |  |
| Ascertainment of exposure (⋆) | Low risk (⋆) | It is a retrospective chart review performed for patients  attending the CNR-JGH clinic between November 2009 and March 2015. |
| Demonstration that outcome of interest was not present at start of study (⋆) | Low risk (⋆) | Assessment at each visit was performed (PCR, albumin level and weight loss, quality of life) |
| Specify how comparability is assessed - main confounder: T-stage (⋆) | N.A. | Single retrospective group |
| Specify how comparability is assessed - other confounders: site (⋆) | N.A. | Single group |
| Assessment of outcome (⋆) | Low risk (⋆) | Values such as PCR, albumin level and weight loss were collected for all patients. Quality of life scores were obtained in over 90%, 6MWT in 325 patients. |
| Adequacy of follow-up length (⋆) | Low risk (⋆) | Six weeks passed between the 1^st^ and 3^rd^ visit. |
| Adequacy of follow-up rate (⋆) | High risk | The reduction in number of patients between the 1^st^ and 3^rd^ visits was 66% |

**Yoon, 2015**

**Risk of bias table**

| **Bias** | **Authors' judgement** | **Support for judgement** |
| --- | --- | --- |
| Representativeness of exposed cohort (⋆) | High Risk | No clear patient enrolment. Cancer care coordinator screened the potential participants. |
| Selection of non-exposed cohort (⋆) | N.A. |  |
| Ascertainment of exposure (⋆) | Low risk (⋆) | Prospective study. Acupuncture treatments were administered by a qualified acupuncturist at his private practice. Intervention well described in the methods. |
| Demonstration that outcome of interest was not present at start of study (⋆) | Low Risk (⋆) | All patients were evaluated before the start of the acupuncture intervention to establish a baseline. Main outcome: The BIA measurements for each time point were standardized to baseline measures and calculated as percentage change. |
| Specify how comparability is assessed - main confounder: T-stage (⋆) | N.A. | Single group |
| Specify how comparability is assessed - other confounders: site (⋆) | N.A. | Single group |
| Assessment of outcome (⋆) | High risk (⋆) | Low risk for outcomes such as BIA and Body mass index. High risk for self-assessment questionnaires as VAS appetite and SNAQ |
| Adequacy of follow-up length (⋆) | Low Risk (⋆) | All patients completed the study for the 8-weeks length |
| Adequacy of follow-up rate (⋆) | Low Risk (⋆) | No subject withdrew from the study. |

**Yuliatun, 2019**

**Risk of bias table**

| **Bias** | **Authors' judgement** | **Support for judgement** |
| --- | --- | --- |
| Representativeness of exposed cohort (⋆) | High risk | Few (7) patients with the same cancer (breast) |
| Selection of non-exposed cohort (⋆) | N.A. |  |
| Ascertainment of exposure (⋆) | Low risk (⋆) | Prospective study. Acupuncture treatments were administered by a qualified acupuncturist at Hospital. Intervention well described in the methods. |
| Demonstration that outcome of interest was not present at start of study (⋆) | Low risk (⋆) | All patients were evaluated before the start of the acupuncture intervention to establish a baseline. Main outcome: The BIA measurements for each time point were standardized to baseline measures and calculated as percentage change. |
| Specify how comparability is assessed - main confounder: T-stage (⋆) | N.A. |  |
| Specify how comparability is assessed - other confounders: site (⋆) | N.A. |  |
| Assessment of outcome (⋆) | Low risk (⋆) | BMI, FAACT free mass and FAACT mass were calculated and their differences before and after the intervention were collected |
| Adequacy of follow-up length (⋆) | Low risk (⋆) | 8 sessions every 2 days. |
| Adequacy of follow-up rate (⋆) | Low risk (⋆) | All patients were evaluated before and after. |

**Buonaccorso, 2023**

**Risk of bias table**

| **Bias** | **Authors' judgement** | **Support for judgement** |
| --- | --- | --- |
| Representativeness of exposed cohort (⋆) | Low risk (⋆) | The study included a convenient sample of 30 consecutive cancer patients in a 900-bed public Comprehensive Clinical Cancer Center. |
| Selection of non-exposed cohort (⋆) | N.A. | + |
| Ascertainment of exposure (⋆) | Low risk (⋆) | Histologically confirmed cancer diagnosis and presence of refractory cachexia and cachexia (assessed by the guidelines of the European Society for Clinical Nutrition and Metabolism -ESPEN3-5 guidelines and the Malnutrition Universal Screening Tool (MUST)) |
| Demonstration that outcome of interest was not present at start of study (⋆) | Low risk (⋆) | The primary outcome is feasibility. Secondary outcomes are Quality of life ad Acceptability. |
| Specify how comparability is assessed - main confounder: T-stage (⋆) | N.A. | N.A. |
| Specify how comparability is assessed - other confounders: site (⋆) | N.A. | N.A. |
| Assessment of outcome (⋆) | Low risk (⋆) | The completion rate was assessed for each component to evaluate the feasibility of psycho-educational intervention combined with rehabilitative intervention among dyads.  Adherence to psycho-educational and physiotherapists meetings was registered and adherence to home exercise sessions was reported by patients using the activity diary.  High Risk for Quality of Life. |
| Adequacy of follow-up length (⋆) | Low risk (⋆) | 8 weeks follow up |
| Adequacy of follow-up rate (⋆) | Low risk (⋆) | Feasibility was the primary outcome (High risk for secondary outcomes) |
