# Supplementary material for: Cancer cachexia: A scoping review on non-pharmacological interventions
Source: Asia Pac J Oncol Nurs. 2024 Mar 12;11(5):100438. doi: 10.1016/j.apjon.2024.100438 (PMC11107192; doi:10.1016/j.apjon.2024.100438)
Supplement: Multimedia component 3 [file mmc3.docx]

**Appendix – Table 1. Risk of bias assessment of randomized studies.** For randomized studies, risk of bias was assessed using the RoB2 tool^13^. The effect of assignment to intervention has been estimated by an intention-to-treat (ITT) analysis.

**Kapoor, 2017**

| **Methods** | Randomized controlled trial |
| --- | --- |
| **Participants** | 63 female patients with advanced cancer attending palliative clinics, with symptoms of cachexia |
| **Interventions** | 30 intervention vs 33 control group |
| **Outcomes** | Anthropometrics, Nutritional status, Physical Activity Level, Quality of life |

| **Domain 6. Overall Bias** | | High risk | |
| --- | --- | --- | --- |
| **Domain 1. Randomization process** | | | |
| **Signalling questions** | **Response** | **Risk-of-bias judgement** | **Justification** |
| **1.1 Was the allocation sequence random?** | Y | Low Risk | “They were allocated study codes, and a randomization sheet was generated using nQuery software (7.0 version)” |
| **1.2 Was the allocation sequence concealed until participants were enrolled and assigned to interventions?** | PY |  | “This could not be a bias because concealment and  randomization were practiced throughout the study.” |
| **1.3 Did baseline differences between intervention groups suggest a problem with the randomization process?** | PN |  | Dietary intake, fat mass, quality of life scores showed statistically significant baseline differences. Nevertheless, the small number of differences identified as ‘statistically significant’ may be considered compatible with chance. |
| **Domain 2. Deviations from intended interventions** | | | |
| **2.1. Were participants aware of their assigned intervention during the trial?** | Y | Low Risk | Open Label in trial protocol, no information on blinding or placebo in manuscript. NCT02350855 |
| **2.2. Were caregivers and people delivering the interventions aware of participants' assigned intervention during the trial?** | Y |  | Open Label in trial protocol, no information on blinding or placebo in manuscript. NCT02350855 |
| **2.3. If Y/PY/NI to 2.1 or 2.2: Were there deviations from the intended intervention that arose because of the trial context?** | PN |  | Only 51% of recruited patients completed the intervention.  Loss to follow-up was mainly driven by deaths, bedridden, travelling and financial difficulties, thus probably not related to the trial context.  Moreover, the authors reported a 41% compliance rate in the intervention group since patients were unable to consume  the advised amount of IAtta. The key reasons reported were anorexia, appetite  loss, mouth sores, and constipation as a result of palliative chemoradiotherapy (for symptom management), toxicity complications, and medications. |
| **2.4 If Y/PY to 2.3: Were these deviations likely to have affected the outcome?** | NA |  | NA |
| **2.5. If Y/PY/NI to 2.4: Were these deviations from intended intervention balanced between groups?** | NA |  | NA |
| **2.6 Was an appropriate analysis used to estimate the effect of assignment to intervention?** | PY |  | Modified Intention-To-Treat analyses |
| **2.7 If N/PN/NI to 2.6: Was there potential for a substantial impact (on the result) of the failure to analyze participants in the group to which they were randomized?** | NA |  | NA |
| **Domain 3. Missing outcome data** | | | |
| **3.1 Were data for this outcome available for all, or nearly all, participants randomized?** | N | High risk | “Out of the 63 patients recruited for the study, 51% completed the intervention.  There were no demographic or clinical differences among the patients who dropped out compared with the ones who finished the study.”  Loss to follow-up was unbalanced (60.0% 18/33 in control group vs 43.3% in intervention group). |
| **3.2 If N/PN/NI to 3.1: Is there evidence that the result was not biased by missing outcome data?** | PN |  | Loss to follow-up was mainly driven by deaths, bedridden, travelling and financial difficulties. These features may be related to quality of life, anthropometrics and behavior characteristics. Thus, a selection of patients due to these causes may affect the resulting estimates.  Loss to follow-up was unbalanced (60.0% 18/33 in control group vs 43.3% in intervention group). |
| **3.3 If N/PN to 3.2: Could missingness in the outcome depend on its true value?** | PY |  |  |
| **3.4 If Y/PY/NI to 3.3: Is it likely that missingness in the outcome depended on its true value?** | PY |  |  |
| **Domain 4. Measurement of the outcome** | | | |
| **4.1 Was the method of measuring the outcome inappropriate?** | N | High risk | Methods, tools and timing of outcome measures reported in the manuscript are valid and sensitive, consistent with declared outcomes and aims. |
| **4.2 Could measurement or ascertainment of the outcome have differed between intervention groups?** | PN |  | Assessment methods and time-points are consistent between groups. |
| **4.3 If N/PN/NI to 4.1 and 4.2: Were outcome assessors aware of the intervention received by study participants?** | Y |  | Open label trial |
| **4.4 If Y/PY/NI to 4.3: Could assessment of the outcome have been influenced by knowledge of intervention received?** | PY |  | Quality of life assessed as self-reported outcome collected by the assessor through a validated questionnaire. |
| **4.5 If Y/PY/NI to 4.4: Is it likely that assessment of the outcome was influenced by knowledge of intervention received?** | PY |  |  |
| **Domain 5. Selection of the reported result** | | | |
| **5.1 Were the data that produced this result analyzed in accordance with a pre-specified analysis plan that was finalized before unblinded outcome data were available for analysis?** | PY | Low risk | Results are reported according to the registered protocol NCT02350855 with the exception of hemoglobin and serum albumin, which were not mentioned in the manuscript. |
| **5.2 Is the numerical result being assessed likely to have been selected, on the basis of the results, from multiple eligible outcome measurements (e.g. scales, definitions, time points) within the outcome domain?** | PN |  | For quality of life domains, a selection of domains were described in depth, yet overall differences were also reported.  Other outcomes were reported as planned in the trial protocol. |
| **5.3**  **Is the numerical result being assessed likely to have been selected, on the basis of the results, from multiple eligible analyses of the data?** | N |  | The authors reported results from analyses consistent with the aims of the study declared in the study protocol. |

**Author Hopkinson, year 2010**

| **Methods** | Cluster-randomized design |
| --- | --- |
| **Participants** | 65 patients with advanced cancer |
| **Interventions** | 35 intervention vs 30 control group |
| **Outcomes** | Interviews pre-and post-intervention for deliverability, acceptability, and patient-perceived effect of intervention; VAS for self-reported eating-related distress and weight-related distress. |

| **Domain 6. Overall Bias** | | **High risk** | |
| --- | --- | --- | --- |
| **Domain 1. Randomization process** | | | |
| **Signalling questions** | **Response** | **Risk-of-bias judgement** | **Justification** |
| **1.1 Was the allocation sequence random?** | Y | Low risk | Simple one-stage cluster randomization |
| **1.2 Was the allocation sequence concealed until participants were enrolled and assigned to interventions?** | Y |  |  |
| **1.3 Did baseline differences between intervention groups suggest a problem with the randomization process?** | N |  | Any differences across the 2 sites were on balance judged less of a threat to the rigor of the study than the risk of contamination effects that would arise with alternative design. |
| **Domain 2. Deviations from intended interventions** | | | |
| **2.1. Were participants aware of their assigned intervention during the trial?** | Y | High risk | Blinding was impossible. |
| **2.2. Were caregivers and people delivering the interventions aware of participants’ assigned intervention during the trial?** | Y |  | The intervention was a nurse-led intervention/consultation. |
| **2.3. If Y/PY/NI to 2.1 or 2.2: Were there deviations from the intended intervention that arose because of the trial context?** | PN |  | 50 out of 65 patients completed the study; non-completion was due to death, decline in clinical conditions, discharge. 71% of patients in the IG and 83% of patients in the CG completed the trial. |
| **2.4 If Y/PY to 2.3: Were these deviations likely to have affected the outcome?** | NA |  |  |
| **2.5. If Y/PY/NI to 2.4: Were these deviations from intended intervention balanced between groups?** | NA |  |  |
| **2.6 Was an appropriate analysis used to estimate the effect of assignment to intervention?** | N |  | Statistical analysis was not appropriate given the small sample size and potential for biased results. |
| **2.7 If N/PN/NI to 2.6: Was there potential for a substantial impact (on the result) of the failure to analyze participants in the group to which they were randomized?** | Y |  |  |
| **Domain 3. Missing outcome data** | | | |
| **3.1 Were data for this outcome available for all, or nearly all, participants randomized?** | N | High risk | Lack of data for the IG at the baseline.  71% of patients in the IG and 83% of patients in the CG completed the trial. |
| **3.2 If N/PN/NI to 3.1: Is there evidence that the result was not biased by missing outcome data?** | N |  | 50 out of 65 patients completed the study; non-completion was due to death, decline in clinical conditions, discharge.  Self-reported scale on perception about weight loss and eating distress. |
| **3.3 If N/PN to 3.2: Could missingness in the outcome depend on its true value?** | Y |  |  |
| **3.4 If Y/PY/NI to 3.3: Is it likely that missingness in the outcome depended on its true value?** | Y |  |  |
| **Domain 4. Measurement of the outcome** | | | |
| **4.1 Was the method of measuring the outcome inappropriate?** | N | High risk | The quantitative scale was self-reported and the qualitative exploration was used to enhance the understanding of VAS and acceptability of MAWE. |
| **4.2 Could measurement or ascertainment of the outcome have differed between intervention groups?** | N |  | Assessment methods and time-points are consistent between groups. |
| **4.3 If N/PN/NI to 4.1 and 4.2: Were outcome assessors aware of the intervention received by study participants?** | PY |  |  |
| **4.4 If Y/PY/NI to 4.3: Could assessment of the outcome have been influenced by knowledge of intervention received?** | PY |  | Quality of life and self-assessment questionnaire potentially also collected a placebo effect. |
| **4.5 If Y/PY/NI to 4.4: Is it likely that assessment of the outcome was influenced by knowledge of intervention received?** | PY |  |  |
| **Domain 5. Selection of the reported result** | | | |
| **5.1 Were the data that produced this result analyzed in accordance with a pre-specified analysis plan that was finalized before unblinded outcome data were available for analysis?** | PY | Low risk | The trial was approved by a local research ethics committee and development approved were gaining before commencing recruitment. |
| **5.2 Is the numerical result being assessed likely to have been selected, on the basis of the results, from multiple eligible outcome measurements (e.g. scales, definitions, time points) within the outcome domain?** | PN |  | The evaluation instrument was a self-reported VAS scale.  The sample size was calculated on the assumption that a difference between control group and MAWE group of 2.5 points in a summary VAS score.  The study was not powered to test the effectiveness of the intervention but was a “best guess” at the number to be recruited per cluster in a follow-on cluster randomized controlled trial. |
| **5.3**  **Is the numerical result being assessed likely to have been selected, on the basis of the results, from multiple eligible analyses of the data?** | N |  | The authors reported results from analyses consistent with the aims of the study declared in the study protocol. |

Faber 2015

| **Methods** | | Exploratory double-blind placebo controlled RCT | |
| --- | --- | --- | --- |
| **Participants** | | 64 newly diagnosed esophageal cancer patients | |
| **Interventions** | | 31 intervention vs 33 control group | |
| **Outcomes** | | Primary outcome: markers for immune function.Secondary outcomes: Body weight, ECOG PS, white blood cell and lymphocyte subset count, inflammatory cytokines, serum PGE_2_, phospholipid fatty acids, pre-albumin, albumin, QoL, dysphagia. | |
|  | |  | |
| **Domain 6. Overall Bias** | |  | |
| **Domain 1. Randomization process** | | | |
| **Signalling questions** | **Response** | **Risk-of-bias judgement** | **Justification** |
| **1.1 Was the allocation sequence random?** | Y | Low risk | Double-blind placebo randomized controlled trial |
| **1.2 Was the allocation sequence concealed until participants were enrolled and assigned to interventions?** | Y |  | “This could not be a bias because concealment and randomization were practiced throughout the study, using a computerized randomization program  after stratification based on their nutritional status”. |
| **1.3 Did baseline differences between intervention groups suggest a problem with the randomization process?** | N |  | No differences  31 pts in IG ( 13 0.5% weight loss and 18% >5% weight loss)  CG 33 pts (16 in 0.5% weight loss and 17> 5% weight loss) |
| **Domain 2. Deviations from intended interventions** | | | |
| **2.1. Were participants aware of their assigned intervention during the trial?** | N | Low risk | Patients in the CG received placebo instead of specific medical food. |
| **2.2. Were caregivers and people delivering the interventions aware of participants' assigned intervention during the trial?** | N |  | Computerized randomization program after the stratification based on nutritional status. Double-blind  The dropout was balanced between groups. 17 out of 64 patients completed the study early due to start of chemotherapy, an increase dysphagia score or other reason.  IG 24 out of 31  CG 23 out of 33 |
| **2.3. If Y/PY/NI to 2.1 or 2.2: Were there deviations from the intended intervention that arose because of the trial context?** | NA |  |  |
| **2.4 If Y/PY to 2.3: Were these deviations likely to have affected the outcome?** | NA |  |  |
| **2.5. If Y/PY/NI to 2.4: Were these deviations from intended intervention balanced between groups?** | NA |  |  |
| **2.6 Was an appropriate analysis used to estimate the effect of assignment to intervention?** | Y |  | All subjects were included in the intention-to-treat analysis.  ANOVA and Mann Whitney test were applied. Correlations were made using the Sperman test. An interim analysis was performed and the sample size confirmed. |
| **2.7 If N/PN/NI to 2.6: Was there potential for a substantial impact (on the result) of the failure to analyze participants in the group to which they were randomized?** | NA |  |  |
| **Domain 3. Missing outcome data** | | | |
| **3.1 Were data for this outcome available for all, or nearly all, participants randomized?** | N | High Risk | Authors say that product compliance was not significantly different between the groups. |
| **3.2 If N/PN/NI to 3.1: Is there evidence that the result was not biased by missing outcome data?** | PN |  |  |
| **3.3 If N/PN to 3.2: Could missingness in the outcome depend on its true value?** | NI |  |  |
| **3.4 If Y/PY/NI to 3.3: Is it likely that missingness in the outcome depended on its true value?** | NI |  |  |
| **Domain 4. Measurement of the outcome** | | | |
| **4.1 Was the method of measuring the outcome inappropriate?** | N | High Risk | The quantitative data on malnourishment were multiple and various.  Quality of life questionnaires were appropriate to measure related eating distress. |
| **4.2 Could measurement or ascertainment of the outcome have differed between intervention groups?** | N |  | Assessment methods and time-points are consistent between groups. |
| **4.3 If N/PN/NI to 4.1 and 4.2: Were outcome assessors aware of the intervention received by study participants?** | PY |  | It is not specified in the article. |
| **4.4 If Y/PY/NI to 4.3: Could assessment of the outcome have been influenced by knowledge of intervention received?** | PY |  | Quality of life questionnaire could potentially always affect assessment of the outcomes. |
| **4.5 If Y/PY/NI to 4.4: Is it likely that assessment of the outcome was influenced by knowledge of intervention received?** | PY |  |  |
| **Domain 5. Selection of the reported result** | | | |
| **5.1 Were the data that produced this result analyzed in accordance with a pre-specified analysis plan that was finalized before unblinded outcome data were available for analysis?** | Y | Low risk | The trial was approved by a local research ethics committee. |
| **5.2 Is the numerical result being assessed likely to have been selected, on the basis of the results, from multiple eligible outcome measurements (e.g. scales, definitions, time points) within the outcome domain?** | N |  | The outcomes were analyzed in total as declared at the beginning pf the study. |
| **5.3**  **Is the numerical result being assessed likely to have been selected, on the basis of the results, from multiple eligible analyses of the data?** | N |  | The authors reported results from analyses consistent with the aims of the study declared in the study protocol. |

Focan 2015

| **Methods** | Prospective RCT feasibility study |
| --- | --- |
| **Participants** | 53 cancer patients with cachexia treated for cancer |
| **Interventions** | 27 intervention vs 26 control group |
| **Outcomes** | Detailed quantitative and qualitative food anamnesis; Quality of life: EORTC QLQ-C30; Mindfulness approach: FFMQ; Satisfaction questionnaire; Body weight, BMI, total daily calories intake, WHO score |

| **Domain 6. Overall Bias** | |  | |
| --- | --- | --- | --- |
| **Domain 1. Randomization process** | | | |
| **Signalling questions** | **Response** | **Risk-of-bias judgement** | **Justification** |
| **1.1 Was the allocation sequence random?** | Y | Low Risk | After informed consent pts were randomized. |
| **1.2 Was the allocation sequence concealed until participants were enrolled and assigned to interventions?** | PY |  | “All subjects benefited from the same clinical and biological evaluation as well as from the same detailed quantitative and qualitative food anamnesis (daily ingested total calories anddistribution of calorie intakes in carbohydrates / lipids / proteins)” |
| **1.3 Did baseline differences between intervention groups suggest a problem with the randomization process?** | PN |  | No significance differences between groups. |
| **Domain 2. Deviations from intended interventions** | | | |
| **2.1. Were participants aware of their assigned intervention during the trial?** | Y | Some concerns | Feasibility trail on quality of life dimension as outcomes and psychological and dietetic intervention in the intervention group. |
| **2.2. Were caregivers and people delivering the interventions aware of participants' assigned intervention during the trial?** | Y |  |  |
| **2.3. If Y/PY/NI to 2.1 or 2.2: Were there deviations from the intended intervention that arose because of the trial context?** | NI |  | Only 12 out of 28 patients in the intervention group completed the study. No data on adherence were shown for the control group. |
| **2.4 If Y/PY to 2.3: Were these deviations likely to have affected the outcome?** | NA |  |  |
| **2.5. If Y/PY/NI to 2.4: Were these deviations from intended intervention balanced between groups?** | NA |  |  |
| **2.6 Was an appropriate analysis used to estimate the effect of assignment to intervention?** | Y |  | Increases in different body parameters were calculated, but the differences between T0 and T1 were processed through SAS software, Student’s t-test, Wilcoxon and Kruskal-Wallis non-parametric tests.  Satisfaction rate of 75% on questionnaires was declared. |
| **2.7 If N/PN/NI to 2.6: Was there potential for a substantial impact (on the result) of the failure to analyze participants in the group to which they were randomized?** | NA |  | NA |
| **Domain 3. Missing outcome data** | | | |
| **3.1 Were data for this outcome available for all, or nearly all, participants randomized?** | N | High risk |  |
| **3.2 If N/PN/NI to 3.1: Is there evidence that the result was not biased by missing outcome data?** | N |  | Loss of follow-up and its reasons were not specified. |
| **3.3 If N/PN to 3.2: Could missingness in the outcome depend on its true value?** | NI |  |  |
| **3.4 If Y/PY/NI to 3.3: Is it likely that missingness in the outcome depended on its true value?** | NI |  |  |
| **Domain 4. Measurement of the outcome** | | | |
| **4.1 Was the method of measuring the outcome inappropriate?** | N | High risk | Methods, tools and timing of outcome measures reported in the manuscript are valid and sensitive, consistent with declared outcomes and aims. |
| **4.2 Could measurement or ascertainment of the outcome have differed between intervention groups?** | PN |  | Assessment methods and time-points are consistent between groups. |
| **4.3 If N/PN/NI to 4.1 and 4.2: Were outcome assessors aware of the intervention received by study participants?** | Y |  | Open-label trial |
| **4.4 If Y/PY/NI to 4.3: Could assessment of the outcome have been influenced by knowledge of intervention received?** | PY |  | Quality of life assessed as self-reported outcome collected by the assessor through a validated questionnaire. |
| **4.5 If Y/PY/NI to 4.4: Is it likely that assessment of the outcome was influenced by knowledge of intervention received?** | PY |  |  |
| **Domain 5. Selection of the reported result** | | | |
| **5.1 Were the data that produced this result analyzed in accordance with a pre-specified analysis plan that was finalized before unblinded outcome data were available for analysis?** | PY | Low risk | Results are reported according to the registered protocol. |
| **5.2 Is the numerical result being assessed likely to have been selected, on the basis of the results, from multiple eligible outcome measurements (e.g. scales, definitions, time points) within the outcome domain?** | N |  | Researchers planned to recruit 429 pts, but only 53 gave consent to the study and 12 completed the intervention. |
| **5.3**  **Is the numerical result being assessed likely to have been selected, on the basis of the results, from multiple eligible analyses of the data?** | N |  | The authors reported results from analyses consistent with the aims of the study declared in the study protocol. |

Grundman 2019, Yoon 2019

| **Methods** | Randomized single-blind pilot study |
| --- | --- |
| **Participants** | 38 Gastrointestinal cancer patients under chemotherapy (no radiotherapy and surgery) |
| **Interventions** | 20 intervention vs 18 control group |
| **Outcomes** | Body composition; Bioelectrical impedance analysis (Fat-free Mass FFM, Intracellular Water ICW, Extracellular Water ECW); Biomarkers Appetite hormones (leptin, ghrelin), systemic inflammation (CRP, TNF-α), nutritional status (prealbumin), and LDH |

| **Domain 6. Overall Bias** | |  | |
| --- | --- | --- | --- |
| **Domain 1. Randomization process** | | | |
| **Signalling questions** | **Response** | **Risk-of-bias judgement** | **Justification** |
| **1.1 Was the allocation sequence random?** | Y | Low Risk | Randomizations were pre-assigned by a statistician. |
| **1.2 Was the allocation sequence concealed until participants were enrolled and assigned to interventions?** | Y |  | “This could not be a bias because concealment and randomization were practiced throughout the study.” |
| **1.3 Did baseline differences between intervention groups suggest a problem with the randomization process?** | PN |  | The 2 groups were comparable in baseline demographics (including cancer diagnosis). |
| **Domain 2. Deviations from intended interventions** | | | |
| **2.1. Were participants aware of their assigned intervention during the trial?** | Y | Some concerns | The intervention is acupuncture. |
| **2.2. Were caregivers and people delivering the interventions aware of participants' assigned intervention during the trial?** | Y |  |  |
| **2.3. If Y/PY/NI to 2.1 or 2.2: Were there deviations from the intended intervention that arose because of the trial context?** | Y |  | IG 15/20 and CG 15/18 for extra transportation arrangements and decreased interest in the study. |
| **2.4 If Y/PY to 2.3: Were these deviations likely to have affected the outcome?** | PY |  |  |
| **2.5. If Y/PY/NI to 2.4: Were these deviations from intended intervention balanced between groups?** | Y |  | 75% of adherence for IG and 83 for control group. |
| **2.6 Was an appropriate analysis used to estimate the effect of assignment to intervention?** | Y |  | Independent Student’s t-test and simple linear regression with p >0.5 for statistical comparison. |
| **2.7 If N/PN/NI to 2.6: Was there potential for a substantial impact (on the result) of the failure to analyze participants in the group to which they were randomized?** | NA |  | NA |
| **Domain 3. Missing outcome data** | | | |
| **3.1 Were data for this outcome available for all, or nearly all, participants randomized?** | Y | Low risk | All patients enrolled in the pilot study  completed the acupuncture intervention and BIA measurements. |
| **3.2 If N/PN/NI to 3.1: Is there evidence that the result was not biased by missing outcome data?** | NA |  |  |
| **3.3 If N/PN to 3.2: Could missingness in the outcome depend on its true value?** | NA |  |  |
| **3.4 If Y/PY/NI to 3.3: Is it likely that missingness in the outcome depended on its true value?** | NA |  |  |
| **Domain 4. Measurement of the outcome** | | | |
| **4.1 Was the method of measuring the outcome inappropriate?** | N | Low risk | Methods, tools and timing of outcome measures reported in the manuscript are valid and sensitive, consistent with declared outcomes and aims. |
| **4.2 Could measurement or ascertainment of the outcome have differed between intervention groups?** | N |  | Assessment methods and time-points are consistent between groups. |
| **4.3 If N/PN/NI to 4.1 and 4.2: Were outcome assessors aware of the intervention received by study participants?** | Y |  |  |
| **4.4 If Y/PY/NI to 4.3: Could assessment of the outcome have been influenced by knowledge of intervention received?** | N |  | Outcomes were objective, such as body composition and fat free mass or extracellular water. |
| **4.5 If Y/PY/NI to 4.4: Is it likely that assessment of the outcome was influenced by knowledge of intervention received?** | NA |  |  |
| **Domain 5. Selection of the reported result** | | | |
| **5.1 Were the data that produced this result analyzed in accordance with a pre-specified analysis plan that was finalized before unblinded outcome data were available for analysis?** | PY | Low risk | Results are reported according to the registered protocol but blinding is not possible. |
| **5.2 Is the numerical result being assessed likely to have been selected, on the basis of the results, from multiple eligible outcome measurements (e.g. scales, definitions, time points) within the outcome domain?** | N |  |  |
| **5.3**  **Is the numerical result being assessed likely to have been selected, on the basis of the results, from multiple eligible analyses of the data?** | N |  | The authors reported results from analyses consistent with the aims of the study declared in the study protocol. |

Kamel 2020

| **Methods** | Single-blind RCT |
| --- | --- |
| **Participants** | 40 patients with pancreatic cancer and cancer-induced cachexia |
| **Interventions** | 20 intervention vs 20 control group |
| **Outcomes** | Mobility, muscle strength, and lean body mass |

| **Domain 6. Overall Bias** | |  | |
| --- | --- | --- | --- |
| **Domain 1. Randomization process** | | | |
| **Signalling questions** | **Response** | **Risk-of-bias judgement** | **Justification** |
| **1.1 Was the allocation sequence random?** | Y | Low Risk | Simple randomization was done by allocating patients with numbers in the IG and CG. |
| **1.2 Was the allocation sequence concealed until participants were enrolled and assigned to interventions?** | Y |  | “Simple randomization was  done by allocating patients with even numbers into the resistance training group and those with odd numbers into the control group. The numbers were placed in opaque and sealed envelopes, and the  participants opened the envelopes for group allocation. Due to the nature of the treatment programme, blinding of patients and physical therapists was not possible. However, blinded isokinetic assessments of muscle strength and Dual-energy X-ray absorptiometry assessments of lean mass were completed” |
| **1.3 Did baseline differences between intervention groups suggest a problem with the randomization process?** | PN |  | No significant differences between demographic characteristics, tumor treatment and surgery. |
| **Domain 2. Deviations from intended interventions** | | | |
| **2.1. Were participants aware of their assigned intervention during the trial?** | Y | Some concerns | Blinding of patients and therapist was not possible. |
| **2.2. Were caregivers and people delivering the interventions aware of participants' assigned intervention during the trial?** | Y |  |  |
| **2.3. If Y/PY/NI to 2.1 or 2.2: Were there deviations from the intended intervention that arose because of the trial context?** | Y |  | IG 17/20 and CG 16/20 due to death, withdrawal and disease progression. |
| **2.4 If Y/PY to 2.3: Were these deviations likely to have affected the outcome?** | PN |  |  |
| **2.5. If Y/PY/NI to 2.4: Were these deviations from intended intervention balanced between groups?** | NA |  |  |
| **2.6 Was an appropriate analysis used to estimate the effect of assignment to intervention?** | NA |  | Sample size was calculated with G* power statistical software.  T-test and Fisher’s exact test to calculate the differences. ANCOVA test. All variables are analyzed with Shapiro-Wilk method. |
| **2.7 If N/PN/NI to 2.6: Was there potential for a substantial impact (on the result) of the failure to analyze participants in the group to which they were randomized?** | NA |  | NA |
| **Domain 3. Missing outcome data** | | | |
| **3.1 Were data for this outcome available for all, or nearly all, participants randomized?** | Y | Low risk | All patients completed  the baseline assessment and post intervention assessments were available for 17 [85%] patients in resistance training group and 16 [80%] patients in the control group.  For patients who died, withdrew or had an  advanced stage of disease-preventing further participation in the study, the statistics was supplemented by their last observed data. For all variables, distribution of standard values was tested using the Shapiro–Wilk method. Levene’s test for variance homogeneity was performed to ensure group homogeneity. |
| **3.2 If N/PN/NI to 3.1: Is there evidence that the result was not biased by missing outcome data?** | NA |  |  |
| **3.3 If N/PN to 3.2: Could missingness in the outcome depend on its true value?** | NA |  |  |
| **3.4 If Y/PY/NI to 3.3: Is it likely that missingness in the outcome depended on its true value?** | NA |  |  |
| **Domain 4. Measurement of the outcome** | | | |
| **4.1 Was the method of measuring the outcome inappropriate?** | N | Low risk | Methods, tools and timing of outcome measures reported in the manuscript are valid and sensitive, consistent with declared outcomes and aims. |
| **4.2 Could measurement or ascertainment of the outcome have differed between intervention groups?** | PN |  | Assessment between groups totally differs depending on intervention; in the control group no exercise regimen was provided, so no physical measurements were calculated.  Common outcomes were measured consistently between arms. |
| **4.3 If N/PN/NI to 4.1 and 4.2: Were outcome assessors aware of the intervention received by study participants?** | PN |  | However, blinded isokinetic assessments  of muscle strength and dual-energy X-ray absorptiometry assessments of lean mass were completed. |
| **4.4 If Y/PY/NI to 4.3: Could assessment of the outcome have been influenced by knowledge of intervention received?** | NA |  |  |
| **4.5 If Y/PY/NI to 4.4: Is it likely that assessment of the outcome was influenced by knowledge of intervention received?** | NA |  |  |
| **Domain 5. Selection of the reported result** | | | |
| **5.1 Were the data that produced this result analyzed in accordance with a pre-specified analysis plan that was finalized before unblinded outcome data were available for analysis?** | PY | Low risk | Results are reported according to the registered protocol, but the blinding is not possible. |
| **5.2 Is the numerical result being assessed likely to have been selected, on the basis of the results, from multiple eligible outcome measurements (e.g. scales, definitions, time points) within the outcome domain?** | N |  |  |
| **5.3**  **Is the numerical result being assessed likely to have been selected, on the basis of the results, from multiple eligible analyses of the data?** | N |  | The authors reported results from analyses consistent with the aims of the study declared in the study protocol and different between arms. |

Molassiotis 2021

| **Methods** | Non-blinded pilot RCT |
| --- | --- |
| **Participants** | 74 Advanced cancer patients and family caregivers, attending the ambulatory at the Royal Brisbane and Women’s Hospital (Australian site), and Haven of Hope Hospital and Shatin Hospital (Hong Kong site). |
| **Interventions** | 34 intervention vs 40 control group |
| **Outcomes** | Feasibility (recruitment, consent rate, retention rate, acceptability of assessment tools).  For patients   - Quality of life: FAACT scale - Nutritional status: PG-SGA-SF (Short Form), 3-day food diary, weight - Eating-related distress: two single-item checklist, on a 1-10 scale   For caregivers   - Anxiety and depression: 14-item HADS - Self-efficacy: 21-item caregiver self-efficacy scale (CaSES) - Caregiver distress: 18-item Caregiver Distress Checklist - Eating-related distress: 19-item eating-related distress checklist |

| **Domain 6. Overall Bias** | |  | |
| --- | --- | --- | --- |
| **Domain 1. Randomization process** | | | |
| **Signalling questions** | **Response** | **Risk-of-bias judgement** | **Justification** |
| **1.1 Was the allocation sequence random?** | Y | Low Risk | The randomization sequence was generated in advance using a computer-generated randomization program. The assignment was sequentially numbered in sealed envelopes. Following recruitment and enrolment of participants and obtaining consent for the study, the research assistants at each site liaised with an independent researcher in the team for randomization allocation. This independent researcher, who was not involved in recruiting patients or delivering the intervention, was responsible for generating the randomization sequence, accessing the next sealed envelope and then advising the research team to which group the patient had been allocated. The research assistants who were responsible for the data collection were the only persons who remained blinded after assignment to the intervention group. |
| **1.2 Was the allocation sequence concealed until participants were enrolled and assigned to interventions?** | Y |  |  |
| **1.3 Did baseline differences between intervention groups suggest a problem with the randomization process?** | N |  | Sample characteristics were well balanced between the two groups and across sites. |
| **Domain 2. Deviations from intended interventions** | | | |
| **2.1. Were participants aware of their assigned intervention during the trial?** | Y | Low Risk | The intervention provided three structured sessions (2–3h) of dietitian direct contact time over a 4-week period, inclusive of telehealth (Australian site only) or telephone follow-ups to monitor, reinforce and adjust goals. |
| **2.2. Were caregivers and people delivering the interventions aware of participants' assigned intervention during the trial?** | PY |  | The research assistants who were responsible for the data collection were the only persons who remained blinded after assignment to intervention group. |
| **2.3. If Y/PY/NI to 2.1 or 2.2: Were there deviations from the intended intervention that arose because of the trial context?** | PN |  | In the Hong Kong setting 8 out of 17 in the IG and 12 out of 25 in CG completed the study.  In the Australia setting 6 out of 17 in the IG and 6 out of 15 in the CG completed the study. |
| **2.4 If Y/PY to 2.3: Were these deviations likely to have affected the outcome?** | NA |  | NA |
| **2.5. If Y/PY/NI to 2.4: Were these deviations from intended intervention balanced between groups?** | NA |  | NA |
| **2.6 Was an appropriate analysis used to estimate the effect of assignment to intervention?** | Y |  | Non-parametric tests were used to conduct inferential analysis owing to the small sample size. Bivariate analysis was done using Chi-Square or Fisher’s exact tests. Differences in outcomes at the two time-points across the two groups were compared using Mann-Whitney U tests. Linear mixed-effects models were used to examine the Group x Time (baseline to final week) interactions on patient and caregiver outcomes. Between-group effect sizes were computed by calculating mean differences of groups with unequal sample size within a pre-post-control design. A p value of 0.05 was set as significant level. |
| **2.7 If N/PN/NI to 2.6: Was there potential for a substantial impact (on the result) of the failure to analyze participants in the group to which they were randomized?** | NA |  |  |
| **Domain 3. Missing outcome data** | | | |
| **3.1 Were data for this outcome available for all, or nearly all, participants randomized?** | PN | High risk | Seventy-four patients and 54 family caregivers participated in the study. Recruitment was challenging, and for every patient agreeing to participate, 14–31 patients had to be screened. The consent rate was 44% in patients and 55% in caregivers. Only half the participants completed the trial’s final assessment. |
| **3.2 If N/PN/NI to 3.1: Is there evidence that the result was not biased by missing outcome data?** | PN |  | In the intervention group (Hong Kong), the dropouts are related to re-hospitalization (2), passed away (3), no mood (1).  In the Australian group, 11 completed the intervention, 5 dropped out due to withdrawal, 1 unable to complete within the study time frame (IG). Two patients did not return the questionnaire.  In the CG 15 completed the intervention, 6 out of 15 did not return the questionnaire. |
| **3.3 If N/PN to 3.2: Could missingness in the outcome depend on its true value?** | PY |  |  |
| **3.4 If Y/PY/NI to 3.3: Is it likely that missingness in the outcome depended on its true value?** | PY |  |  |
| **Domain 4. Measurement of the outcome** | | | |
| **4.1 Was the method of measuring the outcome inappropriate?** | N | Low Risk | Methods, tools and timing of outcome measures reported in the manuscript are valid and sensitive, consistent with declared outcomes and aims. |
| **4.2 Could measurement or ascertainment of the outcome have differed between intervention groups?** | PN |  | Assessment methods and time-points are consistent between groups. |
| **4.3 If N/PN/NI to 4.1 and 4.2: Were outcome assessors aware of the intervention received by study participants?** | PN |  | The research assistants who were responsible for the data collection were the only persons who remained blinded after assignment to the intervention group. Quality-of-life instruments such as FACT were self-perceived instruments. |
| **4.4 If Y/PY/NI to 4.3: Could assessment of the outcome have been influenced by knowledge of intervention received?** | NA |  |  |
| **4.5 If Y/PY/NI to 4.4: Is it likely that assessment of the outcome was influenced by knowledge of intervention received?** | PN |  |  |
| **Domain 5. Selection of the reported result** | | | |
| **5.1 Were the data that produced this result analyzed in accordance with a pre-specified analysis plan that was finalized before unblinded outcome data were available for analysis?** | Y | Low risk | Results were reported according to the registered protocol. |
| **5.2 Is the numerical result being assessed likely to have been selected, on the basis of the results, from multiple eligible outcome measurements (e.g. scales, definitions, time points) within the outcome domain?** | N |  | Outcomes were reported as planned in the trial protocol. |
| **5.3**  **Is the numerical result being assessed likely to have been selected, on the basis of the results, from multiple eligible analyses of the data?** | N |  | The authors reported results from analyses consistent with the aims of the study declared in the study protocol. |

Sim 2022

| **Methods** | RCT |
| --- | --- |
| **Participants** | 58 Gastrointestinal cancer patients |
| **Interventions** | 31 intervention vs 27 control group |
| **Outcomes** | Nutritional status  Body composition through BIA, PG-SGA, body temperature, triceps skinfold thickness, and mid-arm muscle circumference, 3-day 24-hour recall, and concurrent dietary records.  Quality of life  EORTC-QLQ C30 scale.  Nutritional biomarkers  Blood samples were collected at each visit, analyzing hemoglobin, albumin, prealbumin, cholesterol, serum concentrations of tumor necrosis factor-alpha (TNF-α), interleukin-6 (IL-6), and interleukin-8 (IL-8). |

| **Domain 6. Overall Bias** | |  | |
| --- | --- | --- | --- |
| **Domain 1. Randomization process** | | | |
| **Signalling questions** | **Response** | **Risk-of-bias judgement** | **Justification** |
| **1.1 Was the allocation sequence random?** | Y | Some concerns | Cancer patients eligible for the study were randomly allocated to the experimental group and the control group. |
| **1.2 Was the allocation sequence concealed until participants were enrolled and assigned to interventions?** | NI |  | Not specified in the article |
| **1.3 Did baseline differences between intervention groups suggest a problem with the randomization process?** | N |  | Anthropometric and cancer characteristics measures were not significantly different between the two groups. |
| **Domain 2. Deviations from intended interventions** | | | |
| **2.1. Were participants aware of their assigned intervention during the trial?** | Y | Low Risk | Oral nutritional supplements and counselling are in the intervention group vs only counselling in the control group. |
| **2.2. Were caregivers and people delivering the interventions aware of participants' assigned intervention during the trial?** | Y |  |  |
| **2.3. If Y/PY/NI to 2.1 or 2.2: Were there deviations from the intended intervention that arose because of the trial context?** | N |  | Dropouts were a total of 18.  3 out of 18 due to transfers to another hospital, 2 for nausea, poor condition (3), death (5), inclusion in another study.  Dropout for nausea and deterioration could affect the outcomes.  18 in the CG  22 in the IG |
| **2.4 If Y/PY to 2.3: Were these deviations likely to have affected the outcome?** | NA |  |  |
| **2.5. If Y/PY/NI to 2.4: Were these deviations from intended intervention balanced between groups?** | NA |  |  |
| **2.6 Was an appropriate analysis used to estimate the effect of assignment to intervention?** | PY |  | Per protocol analyses were applied. All measurements were expressed as mean ± standard deviation and SPSS program (version 23, 2016, IBM Institute Inc., USA) was used. Baseline anthropometric measures were analyzed using Student’s t-test if the test statistic followed a normal distribution, otherwise Mann-Whitney U test was used to compare the control and intervention groups. Efficacy data were compared using the repeated measured ANOVA test if the test statistic followed a normal distribution. For those data showing skewed distribution, the Mann-Whitney U test was used to determine the group effect, and the Kruskal-Wallis test was used to determine time effect and the interaction between group and time. Comparisons between the mean of the two different weeks were conducted by paired t-test or Wilcoxon signed rank test. Comparisons between the mean of the two groups were conducted by Student’s t-test or Mann-Whitney U test. P-values were determined after Bonferroni corrections for multiple comparisons. |
| **2.7 If N/PN/NI to 2.6: Was there potential for a substantial impact (on the result) of the failure to analyze participants in the group to which they were randomized?** | NA |  | NA |
| **Domain 3. Missing outcome data** | | | |
| **3.1 Were data for this outcome available for all, or nearly all, participants randomized?** | Y | Low risk | During the course of the study, 18 patients dropped out of the study (9 from the control group and 9 from the intervention group) and final analysis was performed in 18 patients in control group and 22 patients in the intervention group. |
| **3.2 If N/PN/NI to 3.1: Is there evidence that the result was not biased by missing outcome data?** | NA |  |  |
| **3.3 If N/PN to 3.2: Could missingness in the outcome depend on its true value?** | NA |  |  |
| **3.4 If Y/PY/NI to 3.3: Is it likely that missingness in the outcome depended on its true value?** | NA |  |  |
| **Domain 4. Measurement of the outcome** | | | |
| **4.1 Was the method of measuring the outcome inappropriate?** | N | Some concerns | Methods, tools and timing of outcome measures reported in the manuscript are valid and sensitive, consistent with declared outcomes and aims. |
| **4.2 Could measurement or ascertainment of the outcome have differed between intervention groups?** | PN |  | Assessment methods and time-points are consistent between groups. |
| **4.3 If N/PN/NI to 4.1 and 4.2: Were outcome assessors aware of the intervention received by study participants?** | Y |  | Not specified in the article but presumably yes. |
| **4.4 If Y/PY/NI to 4.3: Could assessment of the outcome have been influenced by knowledge of intervention received?** | PN |  | Objective outcomes were not influenced by this knowledge (e.g. venous blood or body composition). EORTC quality of life could be influenced. |
| **4.5 If Y/PY/NI to 4.4: Is it likely that assessment of the outcome was influenced by knowledge of intervention received?** | NA |  |  |
| **Domain 5. Selection of the reported result** | | | |
| **5.1 Were the data that produced this result analyzed in accordance with a pre-specified analysis plan that was finalized before unblinded outcome data were available for analysis?** | PY | Low risk |  |
| **5.2 Is the numerical result being assessed likely to have been selected, on the basis of the results, from multiple eligible outcome measurements (e.g. scales, definitions, time points) within the outcome domain?** | PN |  | Outcomes were reported as planned in the trial protocol. |
| **5.3**  **Is the numerical result being assessed likely to have been selected, on the basis of the results, from multiple eligible analyses of the data?** | N |  | The authors reported results from analyses consistent with the aims of the study declared in the study protocol. |

Bagheri 2023

| **Methods** | RCT |
| --- | --- |
| **Participants** | 46 patients with colorectal cancer-induced cachexia |
| **Interventions** | 23 intervention vs 23 control group |
| **Outcomes** | Primary outcomes were muscle strength, lean body mass, nutritional status (assessed with Patient  Generated-Subjective Global Assessment Questionnaire (PG-SGA), and inflammatory markers (high sensitive-C reactive protein, TNF-a, IL-6),  Secondary outcomes werequality of life, serum albumin and total protein.  Quality of life (EORTC-QLQ C30 scale). |

| **Domain 6. Overall Bias** | |  | |
| --- | --- | --- | --- |
| **Domain 1. Randomization process** | | | |
| **Signalling questions** | **Response** | **Risk-of-bias judgement** | **Justification** |
| **1.1 Was the allocation sequence random?** | Y | Low Risk | Stratified block randomization was applied based on BMI and the type of cancer (colon or rectum) via the www.randomization.com web site. |
| **1.2 Was the allocation sequence concealed until participants were enrolled and assigned to interventions?** | Y |  | The statistician will fold the paper containing a randomization number and put them in the envelopes and write the code on them. The envelopes will be kept at the enrollment center. Then, for each qualified patient, the nutritionist randomly picks one of the envelopes after shuffling and allocates patients into control and intervention groups. |
| **1.3 Did baseline differences between intervention groups suggest a problem with the randomization process?** | N |  | Anthropometric and cancer characteristics measures were not significantly different between the two groups. |
| **Domain 2. Deviations from intended interventions** | | | |
| **2.1. Were participants aware of their assigned intervention during the trial?** | Y | Low Risk | It is not possible to blind study participants or staff in this study due to nutritional interventions. Nevertheless, the outcome assessor will be blinded to the group assignment. |
| **2.2. Were caregivers and people delivering the interventions aware of participants' assigned intervention during the trial?** | Y |  |  |
| **2.3. If Y/PY/NI to 2.1 or 2.2: Were there deviations from the intended intervention that arose because of the trial context?** | N |  | Dropouts were a total of 6.  1 death, 4 not adhering to follow up, 1 not adhering to diet.  3 in the CG  3 in the IG |
| **2.4 If Y/PY to 2.3: Were these deviations likely to have affected the outcome?** | NA |  |  |
| **2.5. If Y/PY/NI to 2.4: Were these deviations from intended intervention balanced between groups?** | NA |  |  |
| **2.6 Was an appropriate analysis used to estimate the effect of assignment to intervention?** | PY |  | Intention-to-treat analysis was applied. Adjusted mean changes in primary and secondary outcomes were compared between two groups. |
| **2.7 If N/PN/NI to 2.6: Was there potential for a substantial impact (on the result) of the failure to analyze participants in the group to which they were randomized?** | NA |  | NA |
| **Domain 3. Missing outcome data** | | | |
| **3.1 Were data for this outcome available for all, or nearly all, participants randomized?** | Y | Low risk | 6 out of 46 patients (13%) were lost to follow up, 3 in the intervention group and 3 in the control group |
| **3.2 If N/PN/NI to 3.1: Is there evidence that the result was not biased by missing outcome data?** | NA |  |  |
| **3.3 If N/PN to 3.2: Could missingness in the outcome depend on its true value?** | NA |  |  |
| **3.4 If Y/PY/NI to 3.3: Is it likely that missingness in the outcome depended on its true value?** | NA |  |  |
| **Domain 4. Measurement of the outcome** | | | |
| **4.1 Was the method of measuring the outcome inappropriate?** | N | Low risk | Methods, tools and timing of outcome measures reported in the manuscript are valid and sensitive, consistent with declared outcomes and aims. |
| **4.2 Could measurement or ascertainment of the outcome have differed between intervention groups?** | N |  | Assessment methods and time-points are consistent between groups. |
| **4.3 If N/PN/NI to 4.1 and 4.2: Were outcome assessors aware of the intervention received by study participants?** | N |  | the outcome assessor was blinded to the group assignment. |
| **4.4 If Y/PY/NI to 4.3: Could assessment of the outcome have been influenced by knowledge of intervention received?** | NA |  |  |
| **4.5 If Y/PY/NI to 4.4: Is it likely that assessment of the outcome was influenced by knowledge of intervention received?** | NA |  |  |
| **Domain 5. Selection of the reported result** | | | |
| **5.1 Were the data that produced this result analyzed in accordance with a pre-specified analysis plan that was finalized before unblinded outcome data were available for analysis?** | PY | Low risk | The study protocol was previously published. |
| **5.2 Is the numerical result being assessed likely to have been selected, on the basis of the results, from multiple eligible outcome measurements (e.g. scales, definitions, time points) within the outcome domain?** | N |  | Outcomes were reported as planned in the trial protocol. |
| **5.3**  **Is the numerical result being assessed likely to have been selected, on the basis of the results, from multiple eligible analyses of the data?** | N |  | The authors reported results from analyses consistent with the aims of the study declared in the study protocol. |
